# Exploration of Pyrazole‐Sulfonamide Hybrids: Synthesis, Molecular Modeling, Potential Antimicrobial, DHFR Inhibitor, and Pharmacokinetic Profile

**DOI:** 10.1111/cbdd.70417

**Published:** 2026-10-22

**Authors:** Adhwa A. Alharbi, Manal S. Ebaid, Asmaa L. Alanzy, Adel I. Alalawy, Basma Abbas, Khadra B. Alomari, Roba M. S. Attar, Nashwa M. El‐Metwaly

**Affiliations:** ^1^ Department of Chemistry, College of Science Taibah University Al‐Madinah Al‐Munawarrah Saudi Arabia; ^2^ Department of Chemistry, College of Science Northern Border University Arar Saudi Arabia; ^3^ Department of Chemistry of Natural Compounds, Pharmaceutical and Drug Industries Research Institute National Research Center Cairo Egypt; ^4^ Department of Chemistry, College of Science Qassim University Buraidah Saudi Arabia; ^5^ Department of Biochemistry, Faculty of Science University of Tabuk Tabuk Saudi Arabia; ^6^ Department of Biological Sciences, College of Science University of Jeddah Jeddah Saudi Arabia; ^7^ Department of Physical Sciences, Chemistry Division, College of Science Jazan University Jazan Saudi Arabia; ^8^ Department of Chemistry, Faculty of Science Umm Al‐Qura University Makkah Saudi Arabia

**Keywords:** antibacterial, DFT calculations, DHFR inhibition, docking, pyrazole, sulfonamide

## Abstract

New pyrazolyl sulfonamide conjugates **5**, **6**, and **7** were synthesized by reacting N‐(4‐(hydrazinecarbonyl) aryl) benzenesulfonamide with acetyl acetone, ethyl acetoacetate, and/or 2‐(ethoxymethylene)malononitrile. The DFT computations of the constructed derivatives exhibited comparable bent shapes. The conjugates **5a‐b** and **6a‐b** displayed parallel HOMO‐LUMO structures, whereas the nitro‐derivatives **5c** and **6c** exhibited alternative configurations of their FMOs. Furthermore, the antimicrobial activity was assessed against different bacterial and fungal pathogens. Pyrazole derivatives **5c**, **6b**, **7a**, and **7c** exhibited high activity against *S. aureus* (MIC = 3.125–6.25 μg/mL) and *E. coli* (MIC = 3.125 μg/mL), exceeding the activity of standard antibiotics, with 6b demonstrating the broadest spectrum of activity against both bacteria and fungi. The in vitro DHFR inhibitory effects of the investigated sulfonamides were examined and compared with those of methotrexate. Among the screened derivatives, hybrids **7a‐c** displayed more potent inhibitory activities, thereby determining the effect of substitution patterns on activity. Additionally, the molecular docking of the manufactured analogs against the dihydrofolate reductase enzyme (PDB: 3TZF) was investigated, where hybrid **6a** exhibited the highest activity with a binding score (*S* = −6.5129 kcal/mol), which was greater than that of methotrexate (*S* = −6.4743 kcal/mol). Moreover, pharmacokinetic characteristics were simulated using SwissADME to evaluate drug‐likeness and potential oral bioavailability. All hybrids exhibited beneficial physicochemical properties within acceptable drug‐likeness limits, confirming the pyrazole‐sulfonamide scaffold as an optimization target for future orally active drug candidates with good pharmacokinetic potential.

## Graphical abstract

Nine pyrazole‐sulfonamide hybrids 5, 6, and 7 were synthesized and characterized by IR, NMR, and MS. The DFT computations of the built hybrids presented a comparable non‐planar configuration. Comparable HOMO‐LUMO structures were observed, except for the nitro‐derivatives **5c** and **6c**. Hybrid **6b** exhibited a varied activity range with antifungal activity against *C. albicans* . Hybrids **7a‐c** displayed stronger DHFR inhibitory activities than methotrexate (reference). The SwissADME program revealed approving pharmacokinetic profiles for most hybrids, as orally active nominees.

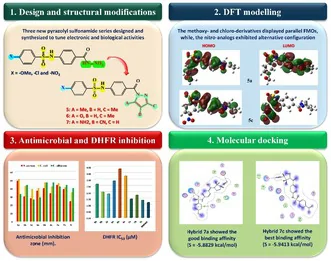

## 1 Introduction

The unyielding emergence of antimicrobial resistance (AMR) is one of the most significant threats to modern medicine and public health worldwide (Alanizi et al. 2024; Naghavi et al. 2024; Harant et al. 2026). The waning efficacy of our existing therapeutic arsenal of antibiotics, particularly versus multidrug‐resistant (MDR) Gram‐negative and positive microorganisms, marks an urgent and unmet need for innovation in the development of novel chemotherapeutic agents with new mechanisms of action (Elshobary et al. 2025; Gajic et al. 2025). This demand has fueled a paradigm shift in antimicrobial drug discovery from classical scaffolds to the rational construction of molecular hybrids (Alkhzem et al. 2022). This approach synergistically combines pharmacophores from distinct bioactive motifs into a single chemical entity to maximize potency, overcome resistance mechanisms, and optimize the overall pharmacological profile (Fu et al. 2017). Against this general strategic backdrop, molecular hybridization of privileged heterocyclic templates with established pharmacophores has been a highly rewarding field of investigation (Sharma, Babu, et al. 2025). The pyrazole core, an example of a five‐membered aromatic heterocycle, is the cornerstone of drug chemistry (Nehra et al. 2026; Ramajayam 2025). In contrast, the sulfonamide pharmacophore (‐SO_2_NH‐) is one of the longest‐standing and most accomplished in chemotherapy history, dating back to the discovery of Prontosil (Gaffer 2019). Sulfonamides and their bioisosteres function as antimetabolites by competing with dihydropteroate synthase (DHFR) for inhibition, a critical enzyme involved in the microorganism pathway to folate biosynthesis (Gaffer et al. 2024). This inhibition drains the pool of tetrahydrofolate (THF), a necessary cofactor in thymidine and purine biosynthesis, leading to eventual bacteriostasis (Rout et al. 2020). However, sulfonamide resistance due to mutant DHFR or enhanced efflux has limited their application as monotherapies. Nevertheless, their proven target validation and high ligand efficiency make them essential building blocks for hybrid molecular design (Ghany et al. 2024; Barman and Goswami 2025; Othman et al. 2020; Dawood et al. 2023). A promising strategy to revive the sulfonamide class and overcome resistance is to combine it with a pyrazole heterocycle to produce novel pyrazole‐sulfonamide hybrids (Ghomashi et al. 2023). Originally, it searches for developing drugs that display dual multi‐target inhibition, if possible, interfering with both dihydropteroate synthase (DHFR) and another diagnostic enzyme, dihydrofolate reductase (DHFR), which tracks in the folate pathway (Bertacine Dias et al. 2018). This concurrent inhibition would have a synergistic antibacterial influence, meaningfully inhibiting the probability of resistance growth. Next, the distinct electronic and steric effect of the pyrazole ring can involve the acidity of the sulfonamide proton, all being well improving the binding affinity to the target enzyme. Moreover, the lipophilicity of the pyrazole core may be rationally tuned to maximize permeation through membranes, particularly across the impermeable outer Gram‐negative bacterial membrane, a significant barrier for most drugs in use early (Sharma, Raju, et al. 2025). The motivation of the present work is to explore the antimicrobial activities of pyrazole‐sulfonamides as antibacterial agents. Both the pyrazole and sulfonamide moieties provide recognized pharmacophores with predictable antibacterial as well as enzyme inhibitory activities. Their combination in a single hybrid molecule is aimed at enhancing biological activity to address the issues of resistance. Their interactions with the dihydrofolate reductase enzyme (DHFR), which is a well‐established antibacterial target, were assessed using molecular modeling, docking, and molecular simulation. In addition, the therapeutic efficacy and drug‐likeness were assessed with pharmacokinetic and ADME/T drug metabolism and excretion modeling. Altogether, this work provides a reasonable rationale for the design of potential lead compounds against drug‐resistant diseases.

## 2 Experimental

### 2.1 General

Melting points (uncorrected) were recorded using a digital Gallenkamp instrument. The FTIR spectra had been verified on a ThermoScientific IS10 spectrometer. The NMR (^1^H at 500‐MHz and ^13^C at 125‐MHz) spectral data were obtained using a JEOL spectrometer in DMSO‐*d*_6_ solutions. Mass analyses have been carried out on a ThermoScientific GC/MS Focus/DSQII (70 eV). A Perkin‐Elmer 2400 analyzer was used for CHN elemental analyses. The starting ethyl 4‐(arylsulfonamido)benzoate compounds **3a‐c** were synthesized in light of the previously published procedure (El‐Sayed et al. 2025) by stirring ethyl 4‐aminobenzoate with aryl sulfonyl chlorides in pyridine at 80°C for an hour.

### 2.2 N‐(4‐(Hydrazinecarbonyl)phenyl)‐4‐substitutedbenzenesulfonamides 4a‐c

To a suspension of each ethyl 4‐(arylsulfonamido)benzoate derivative **3a**, **3b**, or **3c** (12 mmol) in 40 mL ethanol, hydrazine hydrate (0.90 mL, 18 mol) was inserted. The admixture had refluxed for 6 h at 80°C, then cooled to room temperature. The yellow powder that formed has been filtered and crystallized from ethanol to furnish the hydrazide‐sulfonamide analogues **4a**, **4b**, or **4c**, respectively.

#### 2.2.1 N‐(4‐(Hydrazinecarbonyl)phenyl)‐4‐methoxybenzenesulfonamide (**4a**)

Yield = 71.5%, m.p. = 191°C–192°C. IR (cm^−1^): 3335, 3257, 3203 (‐NH_2_ and N‐H), 1658 (C=O), 1606 (C=*N*). ^1^H NMR (*δ*/ppm): 3.80 (s, 3H, OCH_3_), 4.08 (s, 2H, ‐NH_2_), 7.03 (d, *J* = 9.0 Hz, 2H), 7.22 (d, *J* = 8.5 Hz, 2H), 7.47 (d, *J* = 9.0 Hz, 2H), 7.64 (d, *J* = 8.5 Hz, 2H), 9.16 (s, 1H, N‐H), 10.34 (s, 1H, N‐H). MS *m/z* (%) for C_14_H_15_N_3_O_4_S: 321 (22.83, M^+^). Analysis for C_14_H_15_N_3_O_4_S (321.08): Calcd.: C, 52.33; H, 4.71; N, 13.08%. Found: C, 52.23; H, 4.67; N, 13.02%.

#### 2.2.2 4‐Chloro‐N‐(4‐(hydrazinecarbonyl)phenyl)benzenesulfonamide (**4b**)

Yield = 67.8%, m.p. = 178°C–179°C. IR (cm^−1^): 3341, 3295, 3206 (‐NH_2_ and N‐H), 1664 (C=O), 1604 (C=*N*). ^1^H NMR (*δ*/ppm): 4.14 (s, 2H, ‐NH_2_), 7.25 (d, *J* = 8.5 Hz, 2H), 7.56 (d, *J* = 8.5 Hz, 2H), 7.64 (d, *J* = 8.5 Hz, 2H), 7.70 (d, *J* = 8.5 Hz, 2H), 9.24 (s, 1H, N‐H), 10.47 (s, 1H, N‐H). MS *m/z* (%) for C_13_H_12_ClN_3_O_3_S: 325 (17.29, M^+^). Analysis for C_13_H_12_ClN_3_O_3_S (325.03): Calcd.: C, 47.93; H, 3.71; N, 12.90%. Found: C, 47.80; H, 3.77; N, 12.82%.

#### 2.2.3 N‐(4‐(Hydrazinecarbonyl)phenyl)‐4‐nitrobenzenesulfonamide (**4c**)

Yield = 77.2%, m.p. = 217°C–218°C. IR (cm^−1^): 3352, 3300, 3216 (‐NH_2_ and N‐H), 1667 (C=O), 1609 (C=*N*). ^1^H NMR (*δ*/ppm): 4.11 (s, 2H, ‐NH_2_), 7.23 (d, *J* = 8.5 Hz, 2H), 7.68 (d, *J* = 8.5 Hz, 2H), 7.97 (d, *J* = 9.0 Hz, 2H), 8.31 (d, *J* = 9.0 Hz, 2H), 9.18 (s, 1H, N‐H), 10.54 (s, 1H, N‐H). MS *m/z* (%) for C_13_H_12_N_4_O_5_S: 336 (31.17, M^+^). Analysis for C_13_H_12_N_4_O_5_S (336.05): Calcd.: C, 46.43; H, 3.60; N, 16.66%. Found: C, 46.54; H, 3.55; N, 16.75%.

### 2.3 N‐(4‐(3,5‐Dimethyl‐1H‐pyrazole‐1‐carbonyl)phenyl)‐4‐Substitutedbenzenesulfonamides 5a‐c

In a 100 mL RBF, a suspension of each hydrazide‐sulfonamide hybrid **4a**, **4b**, or **4c** (4 mmol) and acetylacetone (0.40 mL, 4 mmol) had been heated for 4 h in ethanol (25 mL). The precipitate formed on cooling to 25°C was collected and crystallized from ethanol to afford the conforming pyrazole‐sulfonamide compounds **5a**, **5b**, or **5c**, respectively.

#### 2.3.1 N‐(4‐(3,5‐Dimethyl‐1H‐pyrazole‐1‐carbonyl)phenyl)‐4‐methoxybenzenesulfonamide (**5a**)

Yield = 82.6%, m.p. = 233°C–234°C. IR (cm^−1^): 3237 (N‐H), 1670 (C=O), 1611 (C=*N*). ^1^H NMR (*δ*/ppm): 2.27 (s, 3H, CH_3_), 2.56 (s, 3H, CH_3_), 3.79 (s, 3H, OCH_3_), 6.11 (s, 1H, pyrazole‐H), 7.04 (d, *J* = 9.0 Hz, 2H), 7.26 (d, *J* = 8.5 Hz, 2H), 7.54 (d, *J* = 9.0 Hz, 2H), 7.73 (d, *J* = 8.5 Hz, 2H), 10.71 (s, 1H, N‐H). ^13^C NMR (*δ*/ppm): 13.02, 13.47, 55.91, 111.62, 114.17 (2C), 119.68 (2C), 124.25, 128.06 (2C), 129.80 (2C), 132.26, 140.11, 141.39, 150.73, 162.54, 165.72. MS *m/z* (%) for C_19_H_19_N_3_O_4_S: 385 (28.37, M^+^). Analysis for C_19_H_19_N_3_O_4_S (385.11): Calcd.: C, 59.21; H, 4.97; N, 10.90%. Found: C, 59.34; H, 4.91; N, 10.82%.

#### 2.3.2 4‐Chloro‐N‐(4‐(3,5‐dimethyl‐1H‐pyrazole‐1‐carbonyl)phenyl)benzenesulfonamide (**5b**)

Yield = 75.8%, m.p. = 241°C–242°C. IR (cm^−1^): 3252 (N‐H), 1673 (C=O), 1608 (C=*N*). ^1^H NMR (*δ*/ppm): 2.22 (s, 3H, CH_3_), 2.58 (s, 3H, CH_3_), 6.09 (s, 1H, pyrazole‐H), 7.21 (d, *J* = 8.5 Hz, 2H), 7.60 (d, *J* = 8.5 Hz, 2H), 7.67 (d, *J* = 8.5 Hz, 2H), 7.75 (d, *J* = 8.5 Hz, 2H), 10.80 (s, 1H, N‐H). ^13^C NMR (*δ*/ppm): 13.08, 13.40, 111.56, 119.51 (2C), 124.17, 128.87 (2C), 129.21 (2C), 129.83 (2C), 138.64, 139.77, 140.48, 141.28, 150.78, 165.67. MS *m/z* (%) for C_18_H_16_ClN_3_O_3_S: 389 (24.66, M^+^). Analysis for C_18_H_16_ClN_3_O_3_S (389.06): Calcd.: C, 55.46; H, 4.14; N, 10.78%. Found: C, 55.62; H, 4.21; N, 10.80%.

#### 2.3.3 N‐(4‐(3,5‐Dimethyl‐1H‐pyrazole‐1‐carbonyl)phenyl)‐4‐nitrobenzenesulfonamide (**5c**)

Yield = 80.1%, m.p. = 265°C–266°C. IR (cm^−1^): 3228 (N‐H), 1678 (C=O), 1613 (C=*N*). ^1^H NMR (*δ*/ppm): 2.24 (s, 3H, CH_3_), 2.58 (s, 3H, CH_3_), 6.23 (s, 1H, pyrazole‐H), 7.27 (d, *J* = 8.5 Hz, 2H), 7.78 (d, *J* = 8.5 Hz, 2H), 8.02 (d, *J* = 9.0 Hz, 2H), 8.36 (d, *J* = 9.0 Hz, 2H), 10.92 (s, 1H, N‐H). ^13^C NMR (*δ*/ppm): 13.04, 13.37, 111.61, 119.58 (2C), 123.46 (2C), 124.04, 128.65 (2C), 129.80 (2C), 140.79, 141.35, 145.17, 149.83, 150.71, 165.94. MS *m/z* (%) for C_18_H_16_N_4_O_5_S: 400 (38.91, M^+^). Analysis for C_18_H_16_N_4_O_5_S (400.08): Calcd.: C, 53.99; H, 4.03; N, 13.99%. Found: C, 53.81; H, 3.95; N, 14.07%.

### 2.4 N‐(4‐(3‐Methyl‐5‐oxo‐4,5‐dihydro‐1H‐pyrazole‐1‐carbonyl)phenyl)‐4‐substituted benzenesulfonamides 6a‐c

To a solution of each hydrazide‐sulfonamide hybrid **4a**, **4b**, or **4c** (4 mmol) in 25 mL ethanol, ethyl acetoacetate (0.52 mL, 4 mmol) was added. The mixture was refluxed for 4 h and then allowed to cool at 20°C–25°C. The crystalline solid was filtered to separate the conforming pyrazole‐sulfonamide hybrids **6a**, **6b**, or **6c**, respectively.

#### 2.4.1 4‐Methoxy‐N‐(4‐(3‐methyl‐5‐oxo‐4,5‐dihydro‐1H‐pyrazole‐1‐carbonyl)phenyl)benzene‐sulfonamide (**6a**)

Yield = 66.4%, m.p. = 269°C–270°C. IR (cm^−1^): 3291 (N‐H), 1664 (C=O), 1630 (C=O). ^1^H NMR (*δ*/ppm): 2.01 (s, 3H, CH_3_), 3.47 (s, 2H, CH_2_), 3.78 (s, 3H, OCH_3_), 7.05 (d, *J* = 9.0 Hz, 2H), 7.28 (d, *J* = 8.5 Hz, 2H), 7.56 (d, *J* = 9.0 Hz, 2H), 7.75 (d, *J* = 8.5 Hz, 2H), 10.63 (s, 1H, N‐H). ^13^C NMR (*δ*/ppm): 16.10, 43.27, 55.88, 113.91 (2C), 119.72 (2C), 122.36, 127.45 (2C), 130.01 (2C), 132.18, 139.84, 161.53, 162.13, 163.40, 167.64. MS *m/z* (%) for C_18_H_17_N_3_O_5_S: 387 (15.76, M^+^). Analysis for C_18_H_17_N_3_O_5_S (387.09): Calcd.: C, 55.81; H, 4.42; N, 10.85%. Found: C, 55.98; H, 4.50; N, 10.74%.

#### 2.4.2 4‐Chloro‐N‐(4‐(3‐methyl‐5‐oxo‐4,5‐dihydro‐1H‐pyrazole‐1‐carbonyl)phenyl)benzene‐sulfonamide (**6b**)

Yield = 63.8%, m.p. = 280°C–281°C. IR (cm^−1^): 3278 (N‐H), 1667 (C=O), 1638 (C=O). ^1^H NMR (*δ*/ppm): 1.98 (s, 3H, CH_3_), 3.51 (s, 2H, CH_2_), 7.23 (d, *J* = 8.5 Hz, 2H), 7.57 (d, *J* = 8.5 Hz, 2H), 7.67 (d, *J* = 8.5 Hz, 2H), 7.78 (d, *J* = 8.5 Hz, 2H), 10.77 (s, 1H, N‐H). ^13^C NMR (*δ*/ppm): 16.13, 43.35, 119.58 (2C), 122.43, 128.81 (2C), 129.25 (2C), 129.92 (2C), 138.36, 139.06, 139.54, 161.67, 163.51, 167.87. MS *m/z* (%) for C_17_H_14_ClN_3_O_4_S: 391 (18.83, M^+^). Analysis for C_17_H_14_ClN_3_O_4_S (391.04): Calcd.: C, 52.11; H, 3.60; N, 10.72%. Found: C, 52.26; H, 3.67; N, 10.80%.

#### 2.4.3 N‐(4‐(3‐Methyl‐5‐oxo‐4,5‐dihydro‐1H‐pyrazole‐1‐carbonyl)phenyl)‐4‐nitrobenzenesulfonamide (**6c**)

Yield = 68.5%, m.p. = 302°C–303°C. IR (cm^−1^): 3302 (N‐H), 1683 (C=O), 1635 (C=O). ^1^H NMR (*δ*/ppm): 2.04 (s, 3H, CH_3_), 3.56 (s, 2H, CH_2_), 7.28 (d, *J* = 8.5 Hz, 2H), 7.76 (d, *J* = 8.5 Hz, 2H), 8.04 (d, *J* = 9.0 Hz, 2H), 8.38 (d, *J* = 9.0 Hz, 2H), 10.91 (s, 1H, N‐H). ^13^C NMR (*δ*/ppm): 16.11, 43.28, 119.33 (2C), 122.60, 123.55 (2C), 128.47 (2C), 129.73 (2C), 139.16, 145.21, 149.96, 161.78, 164.03, 167.58. MS *m/z* (%) for C_17_H_14_N_4_O_6_S: 402 (26.44, M^+^). Analysis for C_17_H_14_N_4_O_6_S (402.06): Calcd.: C, 50.74; H, 3.51; N, 13.92%. Found: C, 50.65; H, 3.46; N, 13.98%.

### 2.5 N‐(4‐(5‐Amino‐4‐cyano‐1H‐pyrazole‐1‐carbonyl)phenyl)‐4‐substitutedbenzene‐sulfonamide 7a‐c

A 100 mL RBF was charged with a solution of each hydrazide‐sulfonamide hybrid **4a**, **4b**, or **4c** (4 mmol) in a mixture of ethanol (20 mL) and DMF (10 mL). After the addition of 2‐(Ethoxymethylene) malononitrile (0.49 g, 4 mmol) and piperidine (0.1 mL), the mixture in each case had been refluxed for 4 h. The conforming pyrazole‐sulfonamide hybrid **7a**, **7b**, or **7c** was precipitated from the hot solution, then filtered by suction and washed with cold ethanol.

#### 2.5.1 N‐(4‐(5‐Amino‐4‐cyano‐1H‐pyrazole‐1‐carbonyl)phenyl)‐4‐methoxybenzenesulfonamide (**7a**)

Yield = 70.2%, m.p. = 222°C–223°C. IR (cm^−1^): 3440, 3305, 3216 (‐NH_2_ and N‐H), 2211 (C ≡ N), 1658 (C=O). ^1^H NMR (*δ*/ppm): 3.80 (s, 3H, OCH_3_), 6.43 (s, 2H, NH_2_), 7.06 (d, *J* = 9.0 Hz, 2H), 7.26 (d, *J* = 8.5 Hz, 2H), 7.52 (d, *J* = 9.0 Hz, 2H), 7.75 (d, *J* = 8.5 Hz, 2H), 8.11 (s, 1H, pyrazole‐H), 10.54 (s, 1H, N‐H). ^13^C NMR (*δ*/ppm): 55.87, 78.72, 114.26 (2C), 115.23, 119.40 (2C), 123.11, 127.83 (2C), 129.64 (2C), 132.18, 140.31, 145.93, 150.59, 163.05, 165.08. MS *m/z* (%) for C_18_H_15_N_5_O_4_S: 397 (36.87, M^+^). Analysis for C_18_H_15_N_5_O_4_S (397.08): Calcd.: C, 54.40; H, 3.80; N, 17.62%. Found: C, 54.28; H, 3.84; N, 17.70%.

#### 2.5.2 N‐(4‐(5‐Amino‐4‐cyano‐1H‐pyrazole‐1‐carbonyl)phenyl)‐4‐chlorobenzenesulfonamide (**7b**)

Yield = 64.3%, m.p. = 209°C–210°C. IR (cm^−1^): 3375, 3310, 3200 (‐NH_2_ and N‐H), 2205 (C ≡ N), 1655 (C=O). ^1^H NMR (*δ*/ppm): 6.50 (s, 2H, NH_2_), 7.28 (d, *J* = 8.5 Hz, 2H), 7.60 (d, *J* = 8.5 Hz, 2H), 7.67 (d, *J* = 8.5 Hz, 2H), 7.81 (d, *J* = 8.5 Hz, 2H), 8.07 (s, 1H, pyrazole‐H), 10.71 (s, 1H, N‐H). ^13^C NMR (*δ*/ppm): 78.90, 115.18, 119.33 (2C), 123.26, 128.81 (2C), 129.28 (2C), 129.58 (2C), 138.71, 139.53, 140.46, 145.74, 150.67, 165.42. MS *m/z* (%) for C_17_H_12_ClN_5_O_3_S: 401 (40.06, M^+^). Analysis for C_17_H_12_ClN_5_O_3_S (401.03): Calcd.: C, 50.81; H, 3.01; N, 17.43%. Found: C, 50.65; H, 2.94; N, 17.30%.

#### 2.5.3 N‐(4‐(5‐Amino‐4‐cyano‐1H‐pyrazole‐1‐carbonyl)phenyl)‐4‐nitrobenzenesulfonamide (**7c**)

Yield = 72.5%, m.p. = 246°C–247°C. IR (cm^−1^): 3391, 3313, 3211 (‐NH_2_ and N‐H), 2210 (C ≡ N), 1664 (C=O). ^1^H NMR (*δ*/ppm): 6.63 (s, 2H, NH_2_), 7.30 (d, *J* = 8.5 Hz, 2H), 7.75 (d, *J* = 8.5 Hz, 2H), 8.03 (d, *J* = 9.0 Hz, 2H), 8.18 (s, 1H, pyrazole‐H), 8.41 (d, *J* = 9.0 Hz, 2H), 10.87 (s, 1H, N‐H). ^13^C NMR (*δ*/ppm): 78.87, 115.28, 119.56 (2C), 122.94, 123.38 (2C), 128.51 (2C), 129.70 (2C), 140.62, 145.25, 146.10, 150.02, 150.65, 165.48. MS *m/z* (%) for C_17_H_12_N_6_O_5_S: 412 (33.96, M^+^). Analysis for C_17_H_12_N_6_O_5_S (412.06): Calcd.: C, 49.51; H, 2.93; N, 20.38%. Found: C, 49.68; H, 3.01; N, 20.23%.

### 2.6 DFT Molecular Modeling

The molecular modeling using density functional theory (DFT) at B3LYP/6–311^++^G(d,p) level included in Gaussian 09 W (Frisch et al. 2009; Becke 1993; Lee et al. 1988; Perdew and Wang 1992) was deployed in inspection of the considered derivatives' three‐dimensional shape without any geometrical constrains, frontier molecular orbitals (FMO's) and electronic features. Frequency calculations were subsequently performed at the same level of theory to confirm that the optimized structures correspond to local minima, as evidenced by the absence of imaginary vibrational frequencies (Karton and de Oliveira 2025). The outcomes have been analyzed using GaussView (Dennington et al. 2009).

### 2.7 Antimicrobial Assay

The antibacterial efficacy of the pyrazole‐sulfonamide analogues was evaluated through assessing their minimum inhibitory concentrations (MIC) as well as inhibition zones (IZ) versus a variety of illnesses. This includes Gram‐positive bacteria like *S. aureus* (ATCC 25923), Gram‐negative bacteria like *E. coli* (ATCC 35218), and fungal strains like *C. albicans* (ATCC 10231). This review evaluated the standards of chloramphenicol, cephalothin, and cycloheximide (see Supporting Information file) (Gaffer et al. 2024; Alhakamy et al. 2020).

### 2.8 DHFR Inhibition Assay

The inhibitory effect of the produced pyrazole‐sulfonamide hybrids against dihydrofolate reductase (DHFR) was assessed using a spectrophotometric enzyme inhibition test. Recombinant human DHFR (or *Escherichia coli* DHFR, depending on the research design) was obtained from a commercial provider such as Sigma‐Aldrich (see Supporting Information file) (Ghomashi et al. 2023).

### 2.9 Molecular Docking Study

The molecular docking studies have been performed using the Molecular Operating Environment (MOE. 2019.0102) software to investigate the interactions of the newly produced pyrazole‐sulfonamide hybrids with two different proteins; a theoretical docking simulation was first conducted using the X‐ray structure. The enzyme DHPS (Dihydroptorate Synthase of Versinia pestis, PDB: ID 3TZF) is wild type (Gaffer et al. 2024). However, energy minimization was accomplished on the protein structure using the default MMFF94x force field until a stable conformation was reached. The active site was defined based on the position of the co‐crystallized ligand, which was also re‐docked into the active site as a validation control, reproducing its experimental binding pose with an RMSD < 2 Å, confirming the reliability of the docking protocol.

## 3 Results and Discussion

### 3.1 Synthesis of Pyrazole‐Sulfonamide Hybrids 5a‐c, 6a‐c, and 7a‐c

The synthetic approach used in the design of pyrazole‐sulfonamide heterocycles **5**, **6**, and **7** is described in Schemes 1 and 2. First, ethyl 4‐aminobenzoate (**2**) was reacted with substituted aryl‐sulphonyl chlorides **1a‐c** to give the corresponding ethyl 4‐(aryl sulfonamido)benzoate derivatives **3a**, **3b**, and **3c**, as described in previous studies (El‐Sayed et al. 2025; Di Magno et al. 2020). The synthetic approach is based on the conversion of ethyl 4‐(arylsulfonamido)‐benzoate derivatives **3a‐c** into the corresponding N‐(4‐(hydrazinecarbonyl)‐phenyl)‐substituted benzene‐sulfonamide compounds **4a‐c** by heating with hydrazine hydrate in ethanol (Scheme 1). The hydrazide products **4a‐c** were valuable intermediate building blocks for assembling three different pyrazole‐containing molecular frameworks.

**SCHEME 1:**
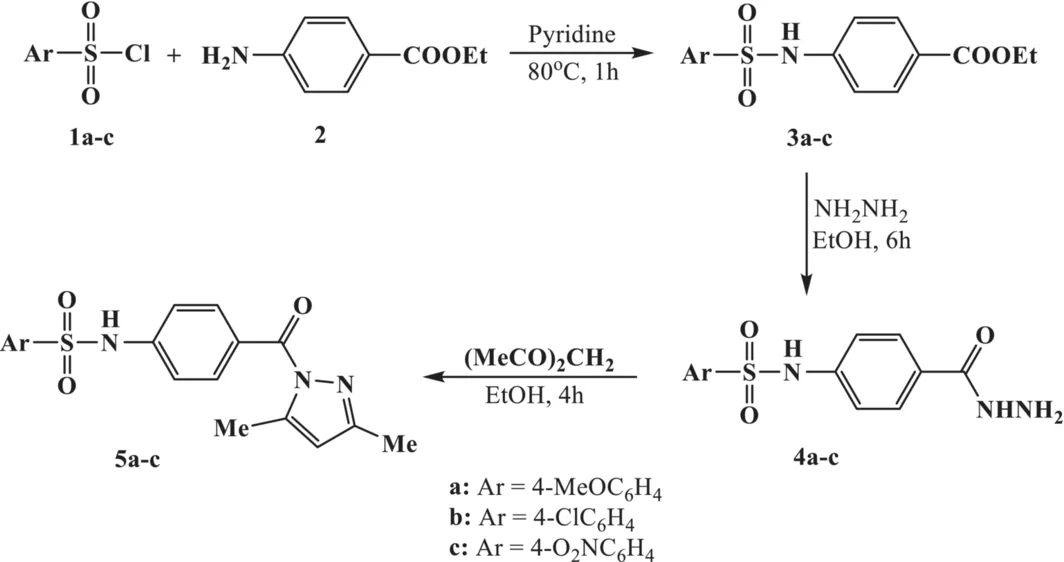
Synthesis of pyrazole‐sulfonamide compounds **5a‐c**.

**SCHEME 2:**
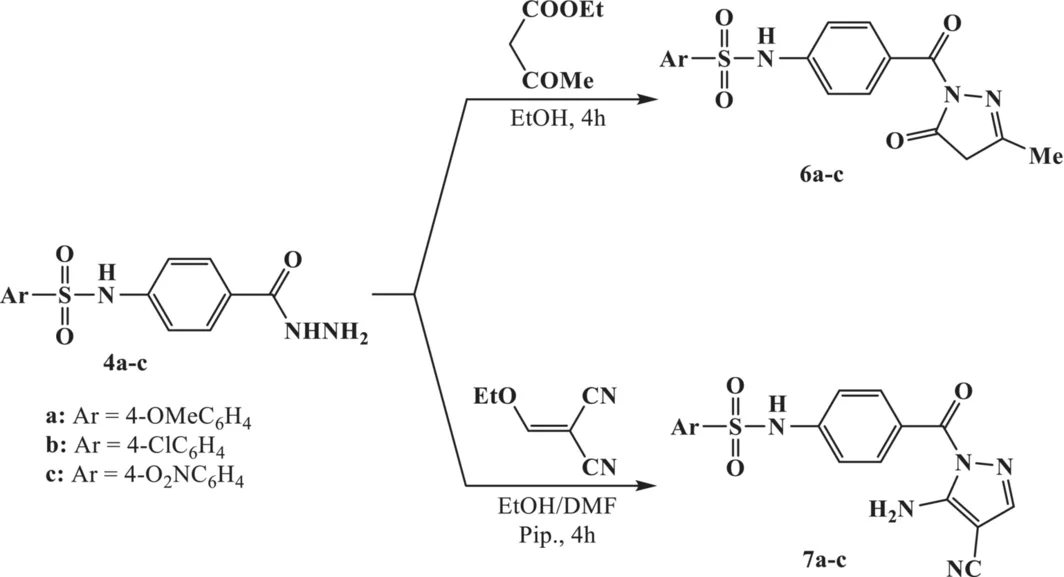
Synthesis of pyrazole‐sulfonamide compounds **6a‐c** and **7a‐c**.

The first class of pyrazole‐sulfonamide conjugates **5a‐c** was obtained in 82.6%, 75.8%, and 80.1% yields by treating hydrazide‐sulfonamide compounds **4a‐c** with acetylacetone in boiling ethyl alcohol. Spectral analyses (IR, NMR, and MS) were used to establish the pyrazole‐sulfonamide **5a‐c** composition. For instance, the IR spectrum of conjugate **5a** announced a stretching band at 3237 cm^−1^ for the N‐H function and at 1670 cm^−1^ for the amidic carbonyl group (C=O). The ^1^H NMR spectrum identified singlet signals at *δ* 2.27, 2.56, and 3.79 ppm, each one integrated for three protons, for the methyl and methoxy groups. The pyrazole's proton was detected as a singlet at *δ* 6.11 ppm. The phenylene protons were observed as doublet signals at *δ* 7.04, 7.26, 7.54, and 7.73 ppm. The proton of the N‐H group was resonated as a singlet at *δ* 10.71 ppm.

The second series of our targeting pyrazole‐sulfonamide hybrids **6a‐c** were synthesized by the reaction of each hydrazide‐sulfonamide compound **4a**, **4b**, or **4c** with ethyl acetoacetate. The reaction was successfully conducted in ethanol under reflux conditions for a duration of 4 h. The formation of pyrazole‐sulfonamide hybrids **6a‐c** was supported by their spectral data and satisfactory elemental analyses (Scheme 2). The FTIR spectrum of hybrid **6b** presented absorptions at 3278, 1667, and 1638 cm^−1^, which evidently suggested the existence of N‐H and carbonyl groups. The ^1^H NMR data displayed the singlet signal for the protons of methyl at *δ* 1.98 ppm (‐CH_3_), methylene at *δ* 3.51 ppm (‐CH_2_‐), and (N‐H) at *δ* 10.77 ppm. The phenylene protons were observed as doublet signals at *δ* 7.23, 7.57, 7.67, and 7.78 ppm.

The reaction of hydrazide‐sulfonamide compound **4a‐c** with 2‐(ethoxymethylene)malononitrile proceeded successfully in hot ethanol/DMF mixture (2:1) containing piperidine to afford the last series of pyrazole‐sulfonamide hybrids **7a**, **7b**, and **7c** in 70.2%, 64.3%, and 72.5% yields, respectively. The IR absorptions (compound **7c**) at 3391, 3313, and 3211 cm^−1^ clearly indicated the presence of amino (‐NH_2_) and N‐H groups. While the recorded absorptions at 2210 and 1664 cm^−1^ were attributed to the nitrile and carbonyl functions, respectively. The ^1^H NMR signals indicated the singlet signal for the protons of amino group at 6.63 ppm (‐NH_2_). The phenylene protons were observed as doublet signals at *δ* 7.30, 7.75, 8.03, and 8.41 ppm. The protons of pyrazole‐C and N‐H group were resonated as singlet signals at *δ* 8.18 and 10.87 ppm.

In summary, the chosen strategy represents a cost‐efficient and modular method for linking the pharmacophore group of the sulfonamide moiety with three different pyrazole‐type structures. The use of hydrazide derivatives **4a‐c** allowed for structural diversity through the alteration of not only the pyrazole scaffold but also the electronic properties of the aryl sulfonamide group. The preparation of compounds **5a‐c**, **6a‐c**, and **7a‐c**, along with their thorough characterization, ensured the generation of a structurally homogeneous chemical series for further theoretical and experimental studies.

### 3.2 DFT Computations

The scrutinized compounds exhibited analogous optimized structures, in which the methoxy, chloro and nitro substituents were coplanar with the phenyl ring bearing them. While, in all conjugates, the sulfonamide oxygen and nitrogen atoms have been deviated from planarity with the benzene sulfonamide ring, e.g., C^2^_(Bsm)_‐C^1^_(Bsm)_‐SO_(Bsm)_‐O^1^S_(Bsm)_ = −18.5° to −24.0°, C^2^_(Bsm)_‐C^1^_(Bsm)_‐SO_(Bsm)_‐O^2^S_(Bsm)_ = 20.8°–27.0° and C^2^_(Bsm)_‐C^1^_(Bsm)_‐SO_(Bsm)_‐NH_(Bsm)_ = 89.1°–93.6°, respectively. Also, the other phenyl has been tilted on the sulfonamide's phenyl and sulfur moieties, where C^1^_(Bsm)_‐SO_(Bsm)_‐NH_(Bsm)_‐C^1^_(Ph)_ = 59.4°–62.1° and SO_(Bsm)_‐NH_(Bsm)_‐C^1^_(Ph)_‐C^2^_(Ph)_ = 47.1°–57.2°, respectively. Moreover, the carbonyl's carbon and oxygen atoms have been moved from the planes of phenyl and pyrazole by ~5.0–6.0 and 12.5°–22.0°, respectively. Correspondingly, the pyrazole ring was slanted on the phenyl nucleus by ~26.7° (Figures 1 and S1; Table S1). In addition, comparison of the geometrical parameters of the hybrids optimized structures with the corresponding values obtained from single‐crystal X‐ray of related compounds (Tao et al. 2020; Laboud et al. 2024) revealed good agreement, with maximum deviations < 0.13 Å for bond lengths and < 16.0° for bond angles (RMSD values = 0.03–0.06 Å and 2.9°–3.8°), respectively. Such insignificant discrepancies could be due to the theoretical DFT calculations are performed for isolated gas‐phase molecule, which does not account for the intermolecular electrostatic interactions operating within the crystal lattice (Sajan et al. 2011) (Tables S2 and S3).

**FIGURE 1:**
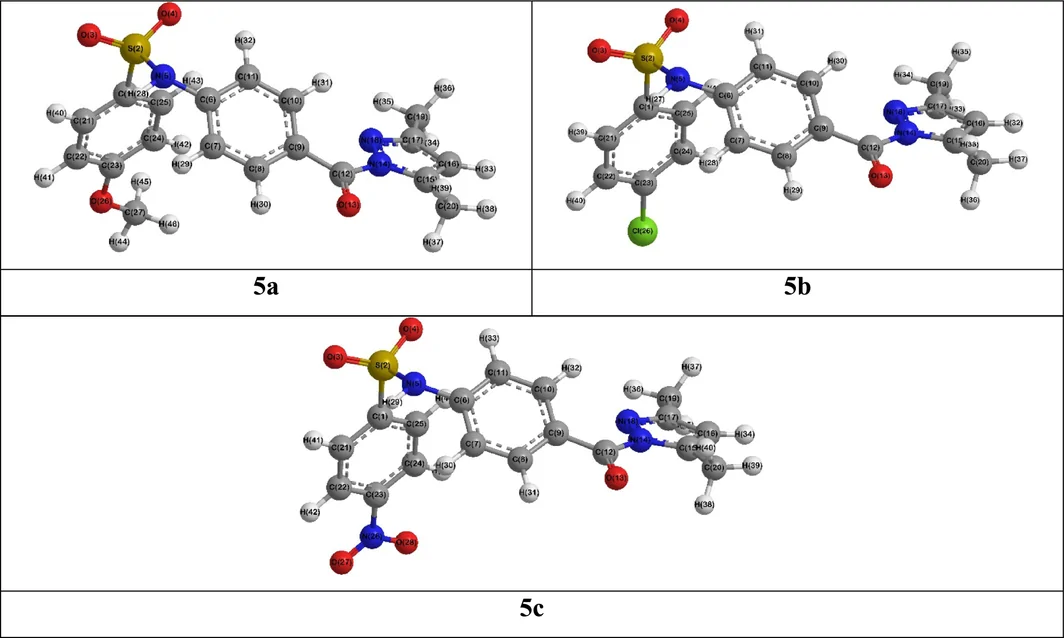
The DFT optimized configurations of hybrids **5a**‐**c**.

In contrast, the frontier molecular orbitals (FMOs), i.e., HOMO and LUMO, skeletons and energies have substantial impact on the molecule's nature to give or gain electrons (Makhlouf et al. 2018; Bouchoucha et al. 2018). The analogues **5a‐b** and **6a‐b** presented comparable of their HOMO, overlaying on the benzene sulfonamide and phenyl carbonyl moieties, and LUMO, wrapping the whole framework (π‐ and π*‐orbitals, respectively). While, the nitro‐analogs **5c** and **6c** exhibited alternative configuration of their FMOs, in which the HOMO was confined to the sulfonamide phenyl carbonyl and nitrobenzene sulfonamide functionals, whereas the LUMO has been spread over the nitrobenzene sulfonamide and entire molecule, respectively. On contrary, the analogs **7a‐c** plots demonstrated comparable configuration of their HOMO, localized on the aminocyanopyrazole nucleus, while their LUMO was extended over the entire framework, excluding the nitro derivative **7c**, in which it was extend over the nitrobenzene sulfonamide group (Figures 2 and S2).

**FIGURE 2:**
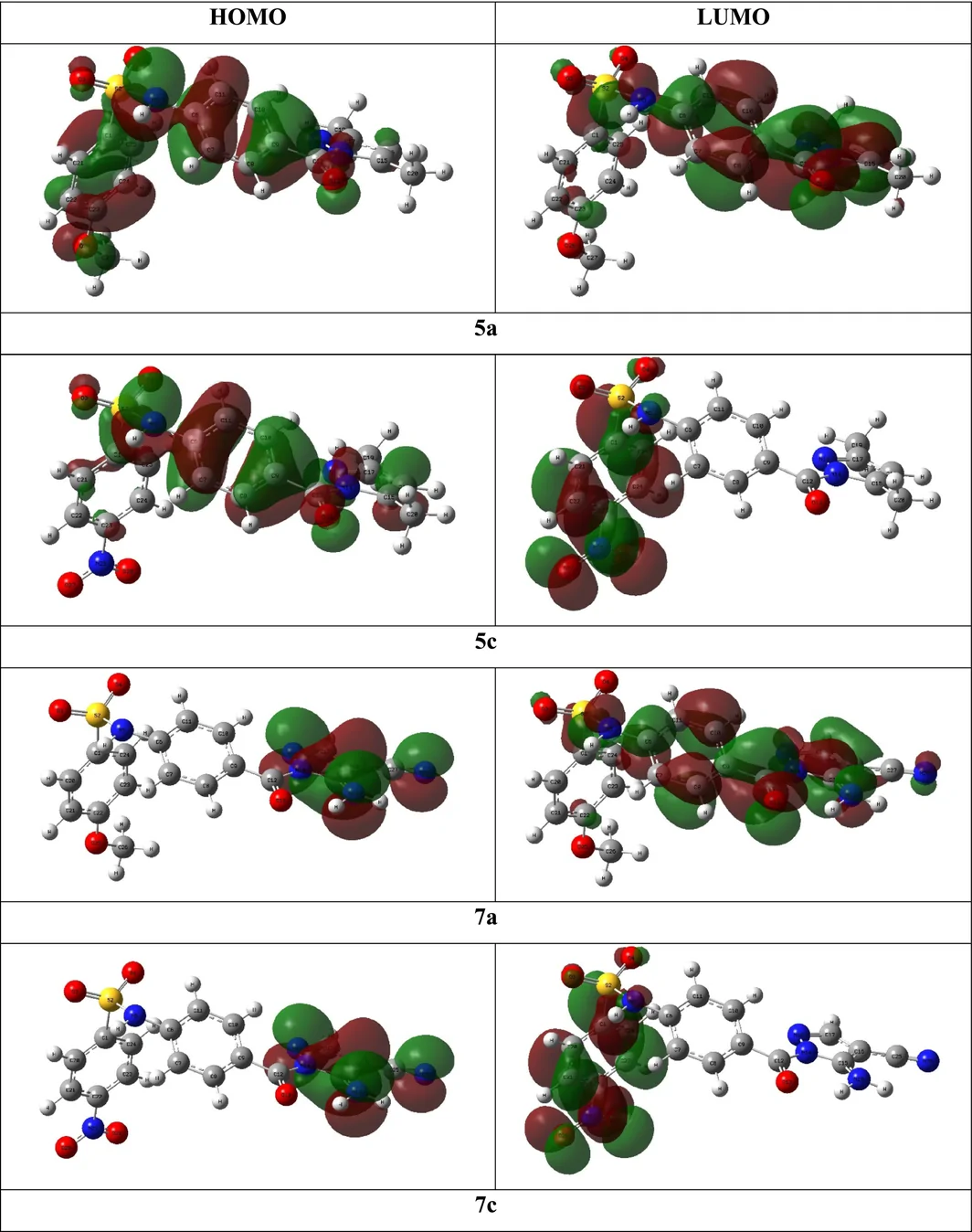
The FMOs plots for pyrazole‐sulfonamide hybrids **5a**, **5c**, **7a**, and **7c**.

Therefore, the inspected hybrids' energies of HOMO (E_H_) ranged from −6.62 to −7.13 eV for **5a** and **6c**, whereas those of LUMO (E_L_) fluctuated from −1.99 to −3.60 eV for **5a** and **7c**, respectively. Moreover, the energy gap (ΔE_H‐L_) was in the 3.19–4.77 eV sector, following the arrangement: **7c** < **6c** < **5c** < **7b** < **7a** < **5a** < **6a** < **5b** < **6b**, indicating that the nitro analogs (**c**) exhibit a reduced gap than the methoxy (**a**) and chloro (**b**) analogs, respectively (Figure 3). Additionally, the FMOs energies were employed in the estimation of several chemical reactivity parameters (Xavier et al. 2015). For instance, both electronegativity and hardness unveiled that compounds **6c** and **6b** showed the topmost values (*χ* = 5.32 and *η* = 2.38 eV), while hybrids **5a** and **7c** donated the lowermost merits (4.30 and 1.60 eV), respectively. Also, the electrophilicity (*ω*) extended from 4.00 (for **5a**) to 8.44 eV (for **7c**), validating the electrophilicity character of these derivatives, *ω* > 1.5 eV, with a greater tendency to donate than receive electrons, owing to electron donating power (*ω* ^+^) < accepting power (*ω* ^−^) (Afolabi et al. 2022) (Table 1).

**FIGURE 3:**
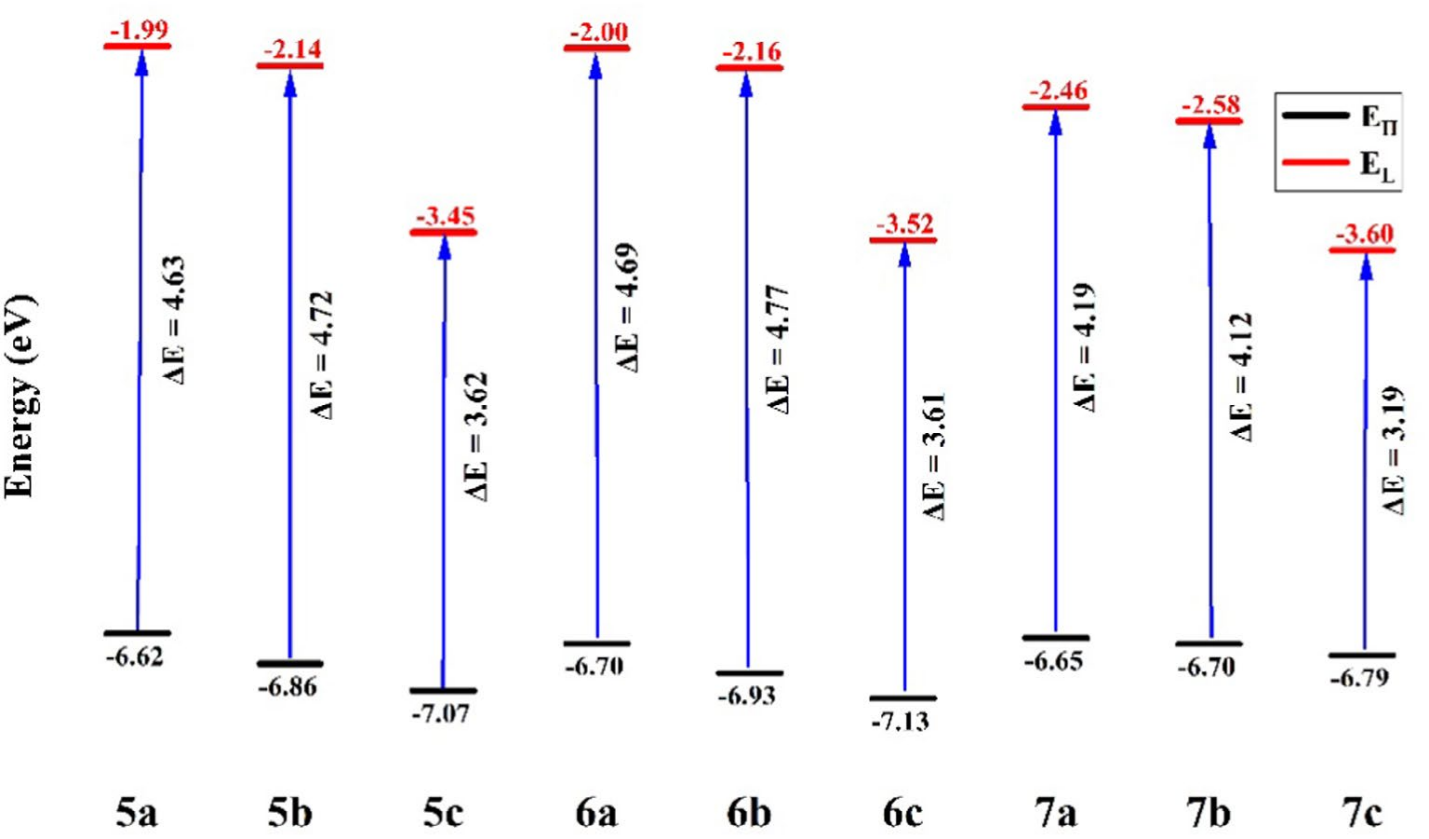
FMOs energies representation of explored hybrids.

**TABLE 1:** FMO's energies and reactivity descriptors (eV) of investigated compounds.

Compound	E_H_	E_L_	ΔE_H‐L_	*χ*	*η*	*δ*	*ω*	*ω+*	*ω−*
**5a**	−6.62	−1.99	4.63	4.30	2.31	0.43	4.00	2.14	6.44
**5b**	−6.86	−2.14	4.72	4.50	2.36	0.42	4.29	2.33	6.83
**5c**	−7.07	−3.45	3.62	5.26	1.81	0.55	7.66	5.25	10.51
**6a**	−6.70	−2.00	4.69	4.35	2.35	0.43	4.03	2.15	6.50
**6b**	−6.93	−2.16	4.77	4.54	2.38	0.42	4.33	2.35	6.90
**6c**	−7.13	−3.52	3.61	5.32	1.81	0.55	7.84	5.41	10.73
**7a**	−6.65	−2.46	4.19	4.56	2.09	0.48	4.96	2.94	7.50
**7b**	−6.70	−2.58	4.12	4.64	2.06	0.49	5.22	3.16	7.80
**7c**	−6.79	−3.60	3.19	5.19	1.60	0.63	8.44	6.05	11.24

Additionally, the intramolecular charge‐transfer and electronegativity perhaps were sympathized more from the Mulliken's atomic charges (Bhagyasree et al. 2013). The data indicated that the sulfonyl sulfur atom SO_(Bsm)_ in all the studied conjugates was consistently positively charged (~0.765), while the attached oxygen atoms O^1^S_(Bsm)_ and O^2^S_(Bsm)_ have acquired equal negative charge (~ −0.583), reflecting the robust electron‐withdrawing character of the sulfonyl functional. In contrast, the amidic nitrogen NH_(Bsm)_ exhibited higher negative charge (−0.759), implying its capability to form H‐bond. Moreover, the benzene ring carbons C^1^_(Bsm)_ and C^4^_(Bsm)_ displayed reasonable variations, particularly the C^4^_(Bsm)_ presented large charge alternations depending on substitution, where in the methoxy (**a**) and nitro (**c**) analogs, it had positive charges of 0.439 and 0.271, respectively, while in the chloro derivatives (**b**), it acquired a low negative charge (−0.020). Expectedly, the carbonyl carbon CO and oxygen OC atoms have played roles as electron‐deficient (0.340–0.394) and electron‐rich centers (−0.331 to −0.421). Interestingly, compounds **7a‐c** showed higher values than other hybrids signifying the stronger polarization and electron‐withdrawing effect in these derivatives. The pyrazolyl nitrogen atoms revealed that the N^1^_(Prz)_ carried a small negative charge (−0.064 to −0.153), whereas N^2^_(Prz)_ had more negative charges (−0.175 to −0.246), where it was attached to the electron‐withdrawing carbonyl group. Such asymmetry indicates that pyrazole acted as an electron mediator between donor and acceptor substituents. Comparison of the examined series indicated that compounds **7a‐c** showed the strongest charge redistribution than **6a‐c** and **5a‐c**, respectively, where the cyano substituents improved the electron‐withdrawing effect, while amino groups enhanced the electron‐donation, and so more efficient intramolecular charge transfer (ICT) (Table S4).

Otherwise, the molecular polarizability (α_total_), hyperpolarizabilities (β_total_), and dipole moment (μ) have been assessed (Abraham et al. 2008) to furnish demonstration of the electronic density distribution and softness, which fundamentally impact the intermolecular interactions (Aziz et al. 2022). The conjugates displayed prevalent dipole moment (μ) varied from 3.33 D (**5c**) to 8.00 D (**6a**), suggesting enhanced charge separation in the latter and implying 2.42–5.83 times the reference urea (Ahmed et al. 2008) (Table 2). As well, the hybrids **5a** and **7c** unveiled the minimum and maximum polarizability (α_total_ = 2.40–2.91 × 10^−23^ esu), while the conjugate **6c** disclosed the lowermost first‐order hyperpolarizability, but **7b** demonstrated the uppermost (β_total_ = 1.77–3.90 × 10^−30^ esu). In contrast to urea (Ahmed et al. 2008), the explored conjugates showed better hyperpolarizability, 4.73–10.43 times, which is coherent with the presence of strong push‐pull substituents that stimulate intramolecular charge transfer (ICT) (Table 2).

**TABLE 2:** The dipole moment (μ), polarizability (α_total_), polarizability anisotropy (Δα), and first‐order hyperpolarizability (β_total_) of explored hybrids.

Compound	μ (Debye)	μ/μ_urea_	α_total_ (esu × 10^−23^)	Δα (esu × 10^−24^)	β_total_ (esu × 10^−30^)	β_total_/β_urea_
**5a**	6.48	4.72	2.40	6.97	2.42	6.47
**5b**	3.89	2.84	2.44	7.06	1.86	4.97
**5c**	3.33	2.42	2.60	8.56	2.44	6.52
**6a**	8.00	5.83	2.47	0.12	2.74	7.34
**6b**	5.16	3.76	2.52	0.12	1.87	4.99
**6c**	3.47	2.52	2.68	0.13	1.77	4.73
**7a**	6.08	4.42	2.70	9.60	3.86	10.33
**7b**	5.10	3.71	2.75	6.89	3.90	10.43
**7c**	4.52	3.29	2.91	6.53	3.52	9.41

### 3.3 Antimicrobial Assay

The antimicrobial assay confirmed that pyrazole‐sulfonamide hybrids exhibited broad‐spectrum activity with varying potencies against microbial strains (Table 3). Among the established hybrids, **5c**, **6b**, **7a**, and **7c** of MIC values (3.125–6.25 μg/mL) versus *S. aureus* in comparison to or even improved than the control drugs such as chloramphenicol and cephalothin (MIC = 6.25 μg/mL). Likewise, in the case of *E. coli* , the hybrids **5c**, **6b**, and **7a** showed MIC = 3.125 μg/mL, which showed greater effectiveness than that of the reference antibiotics (MIC = 6.25 μg/mL) over larger inhibition zones, signifying strong antimicrobial activity. Hybrid **6b**, definitely, was observed to possess significant broad‐spectrum activity with MICs = 3.125 μg/mL versus both bacterial strains and 12.5 μg/mL against *C. albicans* with the supreme inhibition zone (47 mm) against fungal growth, which was greater than cycloheximide (6.25 μg/mL, 33 mm). Correspondingly, **6c** and **7c** revealed balanced antibacterial and antifungal activity with MIC = 6.25 μg/mL among tested strains. Hybrids **5a** and **5b**, however, showed weak to moderate activity (MIC 25–50 μg/mL) with smaller inhibition zones, indicating the decisive effect of structural variations on antimicrobial activity. In combination, the results highlight the paramount role that substitution patterns of sulfonamides play in enhancing broad‐spectrum efficiency, with specific hybrids exceptional in the effectiveness and spectrum of action of predictable antibiotics.

**TABLE 3:** In vitro antimicrobial activities of new pyrazole‐sulfonamide hybrids as MIC (μg/mL) and Inhibition zone (mm).

Hybrids	Gram (+ve) bacteria	Gram (−ve) bacteria	Fungi
	*S. aureus*	*E. coli*	*C. albicans*
**5a**	25 (49)	25 (51)	50 (33)
**5b**	50 (30)	25 (43)	50 (44)
**5c**	3.125 (28)	3.125 (39)	25 (26)
**6a**	12.5 (37)	6.25 (40)	50 (29)
**6b**	3.125 (40)	3.125 (47)	12.5 (47)
**6c**	6.25 (34)	6.25 (42)	6.25 (47)
**7a**	3.125 (44)	3.125 (46)	12.5 (42)
**7b**	3.125 (39)	6.25(41)	12.5 (44)
**7c**	3.125 (24)	6.25 (35)	6.25 (40)
**Chloramphenicol**	6.25 (33)	6.25 (38)	—
**Cephalothin**	6.25 (35)	6.25 (40)	—
**Cycloheximide**	—	—	6.25 (33)

Taken together, it can be noticed that there is a clear electronic effect in all three series in the context of antimicrobial results: nitro‐substituted hybrids **5c**, **6c**, **7c** and chloro‐unsubstituted hybrids **6b** and **7a** always show better results compared to methoxy hybrids **5a**, **6a**, which means that the electron‐withdrawing substituent affects membrane permeability/target interaction more than the substitution in the pyrazole core.

### 3.4 DHFR Inhibition Assay

The in vitro inhibition of the pyrazole‐sulfonamide analogues toward DHFR showed definite trends across the three‐structure series, illustrating the effect of substituent and scaffold modifications on the inhibition activities (Table S5 and Figure 4). For the sulfonamide hybrids **5a‐c containing** the dimethyl pyrazole moiety, hybrid **5a** displayed moderate activity (IC_50_ = 2.66 ± 0.07 μM) with the implication of default activity in the absence of any particular electronic or steric features necessary for enhanced stabilization of the enzyme active site. However, the introduction of a chloro‐substituent in hybrid **5b** showed an inhibition activity (IC_50_ = 2.03 ± 0.11 μM), indicating that minor electronic adjustments can produce a dramatic boost in molecular recognition. Meanwhile, the most active hybrid in this series, **5c** (IC_50_ = 1.88 ± 0.39 μM), exhibited nitro substitution, providing maximum electronic effects for enhanced complementarity in the DHFR‐binding, although its activity was slightly less than methotrexate (1.19 ± 0.30 μM). Switching to the sulfonamide hybrids **6a‐c** containing the pyrazolone moiety, a significant drop in inhibition activity was seen in hybrid **6a** (2.91 ± 0.21 μM) and hybrid **6c** (3.29 ± 0.42 μM) was still a modestly active inhibitor, while hybrid **6b** (3.84 ± 0.50 μM) was the least potent molecule of the set. These results propose that the structural modifications in the **6a‐c** series, particularly the incorporation of the pyrazolinone moiety, may introduce steric hindrance or disrupt the planarity required to reduce the enzyme affinity. In addition, the sulfonamide hybrids **7c** containing the amino‐pyrazole moiety revealed auspicious inhibition against DHFR, as all three hybrids possessed activities approaching methotrexate (reference). Hybrid **7a** (1.46 ± 0.18 μM) exhibited enhanced activity, possibly by the combined effect of both cyano and amino functionality groups that enhance their polarity. Similarly, hybrid **7b** (1.79 ± 0.06 μM) was very active and demonstrated that chloro‐substitution provides beneficial hydrophobic character. Moreover, the lead derivative, **7c** (1.37 ± 0.33 μM), was the most potent of the whole dataset, just less active than methotrexate, thus demonstrating that the nitro‐substituent complements very well the cyano and amino pyrazole platform to enhance the inhibition against the DHFR enzyme. Notably, the IC_50_ values of DHFR inhibition for the hybrids **7a‐c** (1.37 ± 0.33–1.79 ± 0.06 μM) are similar but slightly weaker compared to those of the sulfonamide‐pyrazole hybrids described by Othman et al. (2020) (IC_50_ = 0.09 ± 0.91–0.11 ± 1.05 μM). It is evident from the above results that whereas the current series of molecules confirms the pharmacophore model, the structural.

**FIGURE 4:**
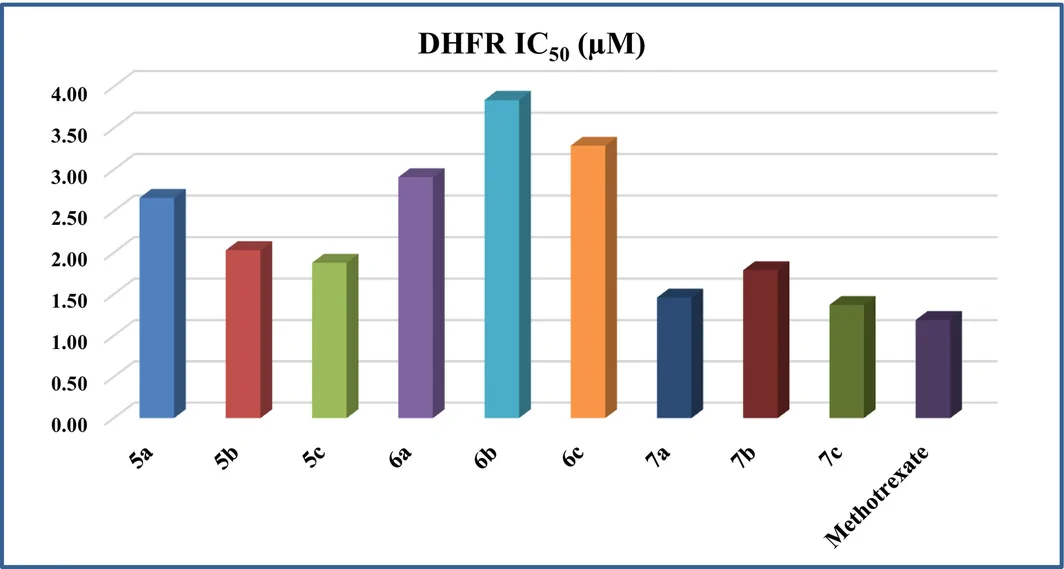
In vitro inhibitory activities of the pyrazole‐sulfonamide hybrids against DHFR enzyme.

In direct comparison of the three pyrazole templates, DHFR inhibition shows the following trend: **7a‐c** (amino‐cyanopyrazole) > **5a‐c** (dimethylpyrazole) > **6a‐c** (pyrazolone), meaning that the presence of a hydrogen‐bond‐donor functional group (amino/nitrile) attached to the pyrazole template is more important for DHFR inhibition activity than the nature of the electron‐withdrawing property of the aryl group of sulfonamide moiety, a phenomenon that does not manifest itself in the antimicrobial screening results, suggesting that bacterial activity and DHFR inhibition are not fully related.

Ultimately, the Multiple Linear Regression (MLR) (Worachartcheewan et al. 2011) included in OriginPro software (OriginLab 2018) has been applied to correlate the antimicrobial inhibition zone (IZ) as well as the DHFR inhibition activity (IC_50_), as dependent variables, with the DFT calculated parameters, E_H_, E_L_, ΔE_H‐L_, *χ*, *η*, *δ*, and *ω*, as independent variables, to explore their effect on the biological activities (Table 4). The resulting models showed high regression coefficients (*R* ^2^) for *S. aureus* (0.9373), *E. coli* (0.9364), *C. albicans* (0.9163), and DHFR inhibition (0.9383), indicating that the combined electronic descriptors captured a substantial proportion of the variation observed within the present series. Among the investigated DFT descriptors, the frontier‐orbital energies (E_H_ and E_L_), HOMO‐LUMO energy gap (ΔE_H‐L_), electronegativity (*χ*), and hardness (*η*) showed the most pronounced contributions to the MLR models, although their relative contributions differ among the tested microorganisms and DHFR inhibition. This variation suggested that the electronic requirements for antimicrobial and enzyme‐inhibitory effects were not identical. However, because of the small dataset and interdependence among these descriptors, the models were considered exploratory rather than predictive. Overall, the MLR analysis provided quantitative support for the relevance of the DFT calculations and indicated that molecular electronic properties contributed to the observed biological activity.

**TABLE 4:** The MLR coefficient of quantum chemical descriptors.

	*S. aureus*	*E. coli*	*C. albicans*	DHFR
**Intercept**	642.59	415.33	−793.75	−24.74
**E**_**H**_	−1949.31	−284.82	2046.39	−286.69
**E**_**L**_	488.04	−207.66	−2678.46	67.61
**ΔE**_**H‐L**_	−691.79	247.12	1667.07	−138.40
***χ***	−1451.64	−518.74	−499.89	−210.53
***η***	−1229.14	−633.89	1454.70	−79.27
***δ***	−433.31	−333.95	754.00	7.12
***ω***	−13.89	7.89	−52.15	−2.45
***R*** ^**2**^	0.9373	0.9364	0.9163	0.9383
**SD**	5.64	3.40	11.83	0.60

### 3.5 Molecular Docking Study

Molecular docking study of the pyrazole‐sulfonamide hybrids provided key insights into their binding profiles and likely modes of interaction within the DHFR enzyme active site (Table S6). Across the sulfonamide hybrids **5a‐c** containing the dimethyl pyrazole moiety (Figure S3), hybrid **5a** showed moderate affinity (−5.2683 kcal/mol) with RMSD 1.9687, held by an H‐donor interaction among the N 5 atom of the sulfonamide group with Glu73 (3.05 Å), supplemented by a π–H interaction between its pyrazole with Leu37 (3.75 Å), reflecting stable but moderate activity. However, hybrid **5b** exhibited a more favorable binding score *S* = −5.8069 kcal/mol and RMSD = 1.5105 with a single but significant H‐bond acceptor interaction amid the O 39 atom of the sulfonamide functional with Lys146 (3.18 Å), which is indicative of improved binding compared to hybrid **5a**. Likewise, hybrid **5c** established a number of stabilizing bindings, such as the O 4 atom of its sulfonamide moiety, which formed hydrogen bonds with Met1 (3.52 Å) and His2 (3.10 Å), along with a π–H stacking between its phenyl ring and Asn180 (4.26 Å), hence possessing a good docking score (*S* = −5.6274 kcal/mol). Most excitingly, through the sulfonamide hybrids **6a‐c** containing the pyrazolone moiety, hybrid **6a** unveiled the best binding score (*S* = −6.5129 kcal/mol over RMSD 1.5461), even superior to methotrexate (reference), with its O 24 atom of the sulfonamide moiety interacting with Thr62 (3.00 Å), the O 33 atom of the amide group interacting with Ser222 (2.87 Å), and its aniline ring interacting with Lys221 through π–H bonding (4.20 Å), all of which together point toward perfect orientation and multiple stabilizing bindings (Figure 5).

**FIGURE 5:**
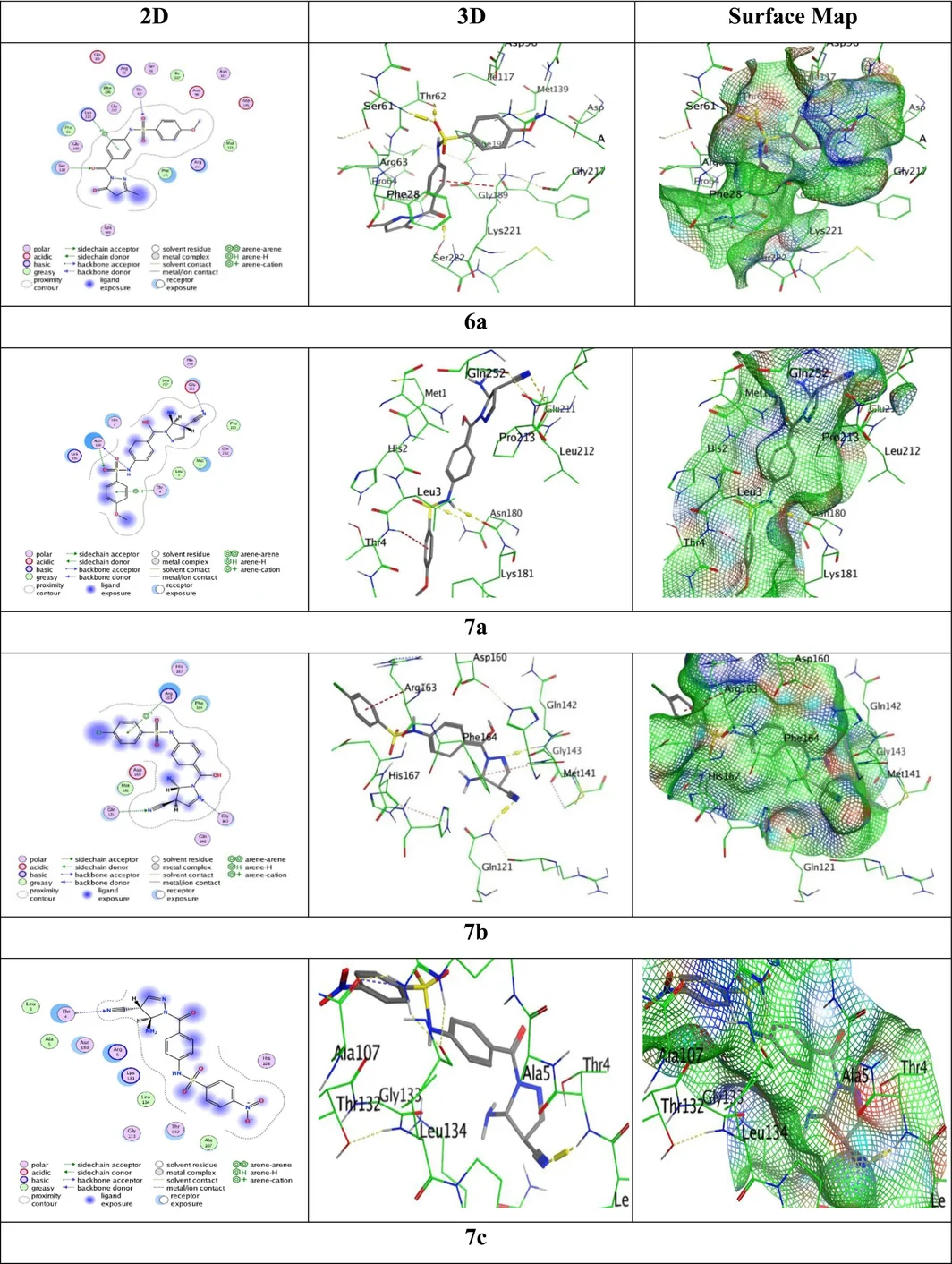
Docking images of hybrids **6a** and **7a‐c** against ID: 3TZF.

In contrast, hybrid **6b** revealed weaker binding (*S* = −4.2781 kcal/mol over RMSD = 1.6556) on the basis of a single hydrogen acceptor binding with Asp11 (3.02 Å), while hybrid **6c** exposed moderate binding score (*S* = −5.1452 kcal/mol) via a hydrogen bond with Asp11 (3.21 Å) (Figure S3). In the sulfonamide hybrids **7‐c** containing the amino‐pyrazole moiety (Figure 5), hybrid **7a** exhibited good binding (*S* = −5.8829 kcal/mol over RMSD = 1.6474) with multiple hydrogen donor and acceptor bindings with Asn180 and Glu211 and a π–H bonding between its Anisyl‐ring and Thr4 (4.86 Å), indicating a multi‐point binding pocket. Meanwhile, hybrid **7b** showed high affinity score (*S* = −5.7566 kcal/mol and RMSD −1.5774) with dual H‐acceptor interactions from both of nitrile and pyrazole groups to Gln121 and Gly143, respectively, and a stabilizing π–H stacking with Arg163 (3.90 Å). Moreover, hybrid **7c** revealed one of the best docking scores (*S* = −5.9413 kcal/mol over RMSD = 1.3003), although it established a single H‐bond between its N 29 atom of the nitrile group and Thr4 (3.25 Å), indicating that the nitrile substitution enhances the binding affinity by optimizing the ligand orientation. For comparison, the reference drug methotrexate presented binding energy (*S* = −6.4743 kcal/mol over RMSD = 1.4710), held by a H‐donor interaction with Glu261 (3.31 Å) and a π–H stacking of its pyrimidine‐ring with Lys260 (4.55 Å) (Figure S3).

It is significant that the ranking in docking of the hybrids **6a** > **7c** ≈ **7a** > **7b** does not completely match the ranking in enzymatic IC_50_ of the hybrids **7c** > **7a** > **7b** > **6a**. This difference may be attributed to the fact that while the rigid docking model is capable of taking into account only the complementarity of the binding pocket, the in vitro experiment additionally considers the effects of solvation and conformational entropy.

### 3.6 Pharmacokinetic Properties

The SwissADME profiling of the pyrazole‐sulfonamide hybrids provided valuable insights into their pharmacokinetic behavior and compliance with drug‐likeness filters (Table S7 and Figure S4I–III). Though the sulfonamide hybrids **5a‐c** contain the dimethyl pyrazole moiety, hybrid **5a**, with M. Wt. = 423.45 g/mol, TPSA = 148.52 Å^2^, and iLogP = 2.22, exhibited adequate solubility, sufficient GI absorption, low BBB penetrability, no Pgp substrate recognition, and full compliance with Lipinski's rule, with a bioavailability grade = 0.55, indicating balanced hydrophilicity and lipophilicity. Nevertheless, hybrid **5b** (Figure S4I) exhibited a slightly higher molecular weight (453.48 g/mol) and TPSA (157.75 Å^2^), showed comparable modest GI absorption and low BBB penetrability, and was also Lipinski‐compliant, which suggests good drug‐like properties despite its slightly polar surface area. Likewise, hybrid **5c** established M. Wt. = 468.45 g/mol, TPSA = 194.34 Å^2^, iLogP = 1.57, and presented one Lipinski violation due to high polarity but still presented reasonable GI absorption and low BBB penetrability; high polarity might impair passive diffusion but enhances solubility. Meanwhile, the sulfonamide hybrids **6a‐c** contain the pyrazolone moiety. Hybrid **6a** (Figure S4II), with a molecular weight of 440.48 g/mol, TPSA of 141.8 Å^2^, and iLogP of 2.26, presented good profiles with moderate GI absorption, little BBB penetrability, no Lipinski's violations, and a bioavailability grade of 0.55 and was one of the most well‐balanced hybrids. Unlike hybrid **6b**, which revealed M. Wt. = 470.5 g/mol, TPSA = 151.03 Å^2^, and iLogP = 2.66, it was slightly heavier but continued to display good qualities, with moderate absorption, little BBB penetrability, and without Lipinski's violations, being pharmacokinetically acceptable. Whereas, hybrid **6c**, exposed to M. Wt. = 485.47 g/mol, TPSA = 187.62 Å^2^, and iLogP = 1.8, exceeded ideal cut‐offs, with a single Lipinski violation, modest GI absorption, and little BBB penetration; its high polarity can potentially inhibit permeability despite good solubility. In the sulfonamide hybrids, **7c** contains the amino‐pyrazole moiety; hybrid **7a** exhibited M. Wt. = 374.42 g/mol, TPSA = 124.79 Å^2^, and iLogP = 2.3 and showed the most balanced profile with good GI absorption, high BBB penetrability, and full Lipinski compliance, revealing its clear potential for central nervous system penetration compared to other analogues. In the meantime, hybrid **7b** displayed M. Wt. = 404.45 g/mol, TPSA = 134.02 Å^2^, and iLogP = 2.46 and maintained good characteristics with sensible absorption, little BBB permeability, no Pgp substrate activity, and full Lipinski compliance, signifying oral appropriateness. Besides, hybrid **7c** discovered M. Wt. = 419.42 g/mol, TPSA = 170.61 Å^2^, iLogP = 1.6, exhibited adequate GI absorption, little BBB penetrability, without Lipinski's violations, and satisfactory bioavailability score; however, its relatively high polarity may decrease CNS penetration. Finally, all of the pyrazole‐sulfonamide hybrids demonstrated similar solubility profiles (reasonable), approving drug‐likeness scores, and absence of Pgp‐mediated efflux liability, investigative of potential for adequate oral bioavailability.

In summary, all the DFT, antimicrobial, DHFR inhibition and docking findings suggest that (i) electron‐withdrawing aryl groups (nitro, chloro) increase antibacterial efficacy and increase DFT‐predicted reactivity (lower HOMO‐LUMO gap), (ii) amino‐cyanopyrazole in hybrids **7a‐c** (Figure S4III) is the best DHFR pharmacophore, and (iii) pyrazolone moiety in hybrids **6a‐c**, although with lower enzyme IC_50_ values, provides the best docking position for hybrid **6a**, suggesting the existence of a second binding mode for this compound, which contributes to its potent whole‐cell efficacy. It is this combined interpretation of findings rather than each individual assay alone that makes hybrids **6b**, **7a** and **7c** the most promising leads.

## 4 Conclusion

Nine new various substituted pyrazolyl‐sulfonamide compounds **5**, **6**, and **7** were synthesized and their chemical structures were elucidated based on their compatible spectral analyses (IR, NMR, and MS). The inspected derivatives displayed resemble FMO's constructions. The energy gap (ΔE_H‐L_) designated that the hybrids **7c** and **6b** seized the lowest and highest values, in addition indicated that the nitro conjugates (**c**) have inferior gap than the methoxy (**a**) and chloro (**b**) analogs, respectively. On the other hand, the antimicrobial efficacy study of showed that **6b**, **6c**, **7a**, and **7c** possessed potent and broad‐spectrum antimicrobial properties against *S. aureus* and *E. coli* , with frequent efficacy being superior to those of typical reference drugs. The outstanding effectiveness of the hybrid **6b** against all the examined microorganisms tested indicated that it is good applicant to be additional advanced and optimized as a promising lead for further antimicrobial optimization. Though, the investigated hybrids exhibited differential DHFR inhibitory activity according to their structural features, i.e., hybrids **5a‐c** were weakly active, while the **7a‐c** were strongly active, almost as potent as the standard drug methotrexate. These results high spot on the hybrids **7a‐c** as the most probable for more development as new hopeful DHFR inhibitors. Moreover, the docking of the synthesized sulfonamides against the active sites of the dihydrofolate reductase (PDB: 3TZF) decorated their potential as novel new antifolate drugs. Hybrid **6a** was the best binding among the hybrids tested, even better than the reference drug methotrexate, while hybrids **7c** and **7a** disclosed better stabilization in the active pockets of the enzyme. Such findings suggest that structure optimization around the nitrile and sulfonamide moieties may result in even higher inhibitory activity and pyrazole‐sulfonamide hybrids as promising scaffolds for future dihydrofolate reductase inhibitors development. Although the current study shows trends in structure activity relationships among DFT, antimicrobial, DHFR inhibition, and docking studies, cytotoxicity evaluation using a normal mammalian cell line was not included in this study and would be the natural progression for determining the therapeutic index of the pyrazole sulfonamide framework before moving forward with further preclinical testing. Moreover, SwissADME pharmacokinetic analysis revealed that all hybrids demonstrated sensible gastrointestinal absorption, no Pgp‐substrate performance, and dependable bioavailability grads = 0.55, as well obeying the Lipinski's rule of five. These obtained results confirm that pyrazole‐sulfonamide scaffolds possess promising pharmaceutical properties susceptible to additional optimization as orally bio‐available drugs. Future work will address two gaps that exist in the current database: studies of enzyme kinetics (Lineweaver‐Burk method), to verify whether the hybrids **7a‐c** exhibit competitive inhibition activity toward DHFR enzyme based on their docked positions, and screening for cytotoxicity on a non‐pathogenic mammalian cell line to determine the selectivity index for the lead hybrids **6b**, **7a**, and **7c**.

## Author Contributions

**Adhwa A. Alharbi:** investigation, validation, methodology, formal analysis. **Manal S. Ebaid:** formal analysis, resources, data curation, writing – review and editing. **Asmaa L. Alanzy:** writing – review and editing, validation, methodology, investigation. **Adel I. Alalawy:** validation, formal analysis, data curation. **Basma Abbas:** data curation, resources, writing – review and editing, writing – original draft, visualization. **Khadra B. Alomari:** resources, software, methodology, validation, investigation. **Roba M. S. Attar:** validation, software, data curation, writing – original draft. **Nashwa M. El‐Metwaly:** writing – review and editing, project administration.

## Funding

This research work was funded by Umm Al‐Qura University, Saudi Arabia under grant number: (26UQU4350527GSSR07).

## Ethics Statement

The authors have nothing to report.

## Consent

All authors participated directly in the current research work.

## Conflicts of Interest

The authors declare no conflicts of interest.

## Supporting information


**Table S1:** The DFT dihedral angle data of the investigated compounds.
**Table S2:** The DFT bond length data of the investigated compounds.
**Table S3:** The DFT bond angle data of the investigated compounds.
**Table S4:** Mulliken's atomic charges of explored hybrids.
**Table S5:** In vitro inhibitory activities of the synthesized pyrazole‐sulfonamide hybrids against DHFR enzyme.
**Table S6:** Docking results of the synthesized pyrazole‐sulphonamide hybrids with PDB: 3TZF.
**Table S7:** SwissADME results of the synthesized pyrazole‐sulphonamide hybrids.
**Figure S1:** The optimized structures of derivatives **6a‐c** and **7a‐c**.
**Figure S2:** The 3D‐plot of FMO's for **5b**, **6a‐c** and **7c** compounds.
**Figure S3:** Docking images of hybrids **5a‐c**, **6b**, **6c**, and methotrexate with ID: 3TZF.
**Figure S4: (I; 5a‐5c: II; 6a‐6c: III; 7a‐7c)** Radar chart and boiled egg of the synthesized pyrazole‐sulphonamide hybrids.

## Data Availability

The data that support the findings of this study are available on request from the corresponding author. The data are not publicly available due to privacy or ethical restrictions.
