# Supplementary material for: Exploration of Pyrazole‐Sulfonamide Hybrids: Synthesis, Molecular Modeling, Potential Antimicrobial, DHFR Inhibitor, and Pharmacokinetic Profile
Source: Chem Biol Drug Des. 2026 Oct 22;108(4):e70417. doi: 10.1111/cbdd.70417 (PMC13691284; doi:10.1111/cbdd.70417)
Supplement: Supplementary file 1 [file CBDD-108-e70417-s001.docx]

**Supplementary file**

**Antimicrobial Activity**

***Antibacterial activity***

According to inhibition zones method bacterial cultures were matured in nutrient agar media at 30 °C. After 16 h of growth, each microorganism, at a concentration of 10^8^cells/mL was put on the surface of Mueller-Hinton agar plates using sterile cotton. Inoculated disc was tested by millimeter ruler, after incubation, the diameters of inhibition zones (IZ) were measured.

***Antifungal activity***

The dynamic inoculum for the assays was set up by exchanging numerous spore loops from the stock cultures to check the containers of sterile distilled water that were upset and diluted to obtain an optical thickness corresponding to 2.0×10^5^ spore/mL inoculum of 0.1 spore/mL for each penny of suspension, the swab was regularly sponged and the inoculum was dried for 5 min, then a similar strategy was adopted.

***Minimum inhibitory concentration (MIC)testing***

The broth microdilution method was used to determine the MIC of the newly synthesized, pyrazole-sulfonamides analogues against various pathogenic microorganisms. A stock solution of the antimicrobial agent at a known concentration was prepared. Serial two-fold dilutions of the stock solution were performed in the broth medium (Mueller-Hinton broth for bacteria, and RPMI-1640 for fungi) to create a range of concentrations in the microtiter plate wells. Typically, the concentration range spans several orders of magnitude. A standardized inoculum of the test microorganism was prepared by adjusting the turbidity of a bacterial and fungal suspension to a specific McFarland standard. A fixed volume of the standardized inoculum was added to each well of the microtiter plate, ensuring equal distribution. The microtiter 96-well plates were incubated at an appropriate temperature (37°C for bacteria, 30°C for fungi) for 18-24 hours. After incubation, the MIC results were measured visually by inspection of the microtiter plate to identify the lowest concentration of the antimicrobial agent that inhibits the visible growth of the microorganism. Alternatively, a microplate reader was used to measure the optical density (OD) of each well. The MIC is determined as the lowest concentration at which the OD is significantly reduced compared to the growth control well. Chloramphenicol, cephalothin, and cycloheximide were employed as antibiotics “positive controls”.

**DHFR inhibition assay**

The assay mixture contained 1.5 M sodium acetate buffer (pH 6), 0.6 M KCl, 50 µM NADPH, 50 µL dimethylsulphoxide (DMSO) or the same volume of a DMSO solution of test compounds, to a final concentration of between 10^-11^ and 10^-5^ M, and 0.02 units of bovine liver DHFR, in a final volume of 3 mL. After addition of the enzyme, the mixture was incubated at 23 °C for 5 min, and the reaction was started by adding 33 µM dihydro-folic acid. The change in absorbance at 340 nm was followed for 10 min. Results are reported as percent inhibition of enzymatic activity.

**Table S1**. The DFT dihedral angle data of the investigated compounds.

|  | **5a** |  | **5b** |  | **5c** |  | **6a** |  | **6b** |
| --- | --- | --- | --- | --- | --- | --- | --- | --- | --- |
| C(23)-C(24)-C(25)-C(1) | 0.3 | C(23)-C(24)-C(25)-C(1) | 0.4 | C(23)-C(24)-C(25)-C(1) | 0.4 | C(23)-C(24)-C(25)-C(1) | 0.2 | C(23)-C(24)-C(25)-C(1) | 0.4 |
| C(22)-C(23)-O(26)-C(27) | 179.8 | C(22)-C(23)-C(24)-C(25) | 0.3 | C(22)-C(23)-N(26)-O(27) | -1.6 | C(22)-C(23)-O(26)-C(27) | 179.7 | C(22)-C(23)-C(24)-C(25) | 0.3 |
| C(24)-C(23)-O(26)-C(27) | 0.2 | Cl(26)-C(23)-C(24)-C(25) | 179.9 | C(22)-C(23)-N(26)-O(28) | 179.4 | C(24)-C(23)-O(26)-C(27) | 0.2 | Cl(26)-C(23)-C(24)-C(25) | 179.9 |
| C(22)-C(23)-C(24)-C(25) | 0.3 | C(21)-C(22)-C(23)-C(24) | -0.5 | C(24)-C(23)-N(26)-O(27) | 179.0 | C(22)-C(23)-C(24)-C(25) | 0.3 | C(21)-C(22)-C(23)-C(24) | -0.5 |
| O(26)-C(23)-C(24)-C(25) | 179.9 | C(21)-C(22)-C(23)-Cl(26) | 179.9 | C(24)-C(23)-N(26)-O(28) | 0.0 | O(26)-C(23)-C(24)-C(25) | 179.9 | C(21)-C(22)-C(23)-Cl(26) | 179.9 |
| C(21)-C(22)-C(23)-C(24) | -0.5 | C(1)-C(21)-C(22)-C(23) | 0.1 | C(22)-C(23)-C(24)-C(25) | 0.3 | C(21)-C(22)-C(23)-C(24) | -0.5 | C(1)-C(21)-C(22)-C(23) | 0.1 |
| C(21)-C(22)-C(23)-O(26) | 179.9 | C(16)-C(17)-N(18)-N(14) | -0.1 | N(26)-C(23)-C(24)-C(25) | 179.7 | C(21)-C(22)-C(23)-O(26) | 179.9 | C(16)-C(17)-N(18)-N(14) | -0.2 |
| C(1)-C(21)-C(22)-C(23) | 0.0 | C(19)-C(17)-N(18)-N(14) | 179.8 | C(21)-C(22)-C(23)-C(24) | -0.5 | C(1)-C(21)-C(22)-C(23) | 0.1 | C(19)-C(17)-N(18)-N(14) | 179.4 |
| C(16)-C(17)-N(18)-N(14) | 0.0 | C(15)-C(16)-C(17)-N(18) | 0.5 | C(21)-C(22)-C(23)-N(26) | -179.9 | C(16)-C(17)-N(18)-N(14) | -0.2 | C(15)-C(16)-C(17)-N(18) | 0.2 |
| C(19)-C(17)-N(18)-N(14) | 179.8 | C(15)-C(16)-C(17)-C(19) | -179.3 | C(1)-C(21)-C(22)-C(23) | 0.0 | C(19)-C(17)-N(18)-N(14) | 179.4 | C(15)-C(16)-C(17)-C(19) | -179.4 |
| C(15)-C(16)-C(17)-N(18) | 0.5 | N(14)-C(15)-C(16)-C(17) | -0.7 | C(16)-C(17)-N(18)-N(14) | -0.1 | C(15)-C(16)-C(17)-N(18) | 0.3 | N(14)-C(15)-C(16)-C(17) | -0.1 |
| C(15)-C(16)-C(17)-C(19) | -179.4 | C(20)-C(15)-C(16)-C(17) | -179.5 | C(19)-C(17)-N(18)-N(14) | 179.7 | C(15)-C(16)-C(17)-C(19) | -179.3 | O(20)-C(15)-C(16)-C(17) | -178.1 |
| N(14)-C(15)-C(16)-C(17) | -0.7 | C(12)-N(14)-N(18)-C(17) | -175.6 | C(15)-C(16)-C(17)-N(18) | 0.5 | N(14)-C(15)-C(16)-C(17) | -0.2 | C(12)-N(14)-N(18)-C(17) | -172.6 |
| C(20)-C(15)-C(16)-C(17) | -179.5 | C(15)-N(14)-N(18)-C(17) | -0.4 | C(15)-C(16)-C(17)-C(19) | -179.3 | O(20)-C(15)-C(16)-C(17) | -178.2 | C(15)-N(14)-N(18)-C(17) | 0.1 |
| C(12)-N(14)-N(18)-C(17) | -175.9 | C(12)-N(14)-C(15)-C(16) | 175.6 | N(14)-C(15)-C(16)-C(17) | -0.7 | C(12)-N(14)-N(18)-C(17) | -172.9 | C(12)-N(14)-C(15)-C(16) | 172.3 |
| C(15)-N(14)-N(18)-C(17) | -0.4 | C(12)-N(14)-C(15)-C(20) | -5.6 | C(20)-C(15)-C(16)-C(17) | -179.6 | C(15)-N(14)-N(18)-C(17) | 0.0 | C(12)-N(14)-C(15)-O(20) | -9.7 |
| C(12)-N(14)-C(15)-C(16) | 175.9 | N(18)-N(14)-C(15)-C(16) | 0.7 | C(12)-N(14)-N(18)-C(17) | -175.5 | C(12)-N(14)-C(15)-C(16) | 172.6 | N(18)-N(14)-C(15)-C(16) | 0.0 |
| C(12)-N(14)-C(15)-C(20) | -5.3 | N(18)-N(14)-C(15)-C(20) | 179.6 | C(15)-N(14)-N(18)-C(17) | -0.4 | C(12)-N(14)-C(15)-O(20) | -9.4 | N(18)-N(14)-C(15)-O(20) | 178.1 |
| N(18)-N(14)-C(15)-C(16) | 0.7 | C(9)-C(12)-N(14)-C(15) | 167.7 | C(12)-N(14)-C(15)-C(16) | 175.5 | N(18)-N(14)-C(15)-C(16) | 0.1 | C(9)-C(12)-N(14)-C(15) | 161.6 |
| N(18)-N(14)-C(15)-C(20) | 179.5 | C(9)-C(12)-N(14)-N(18) | -17.9 | C(12)-N(14)-C(15)-C(20) | -5.6 | N(18)-N(14)-C(15)-O(20) | 178.1 | C(9)-C(12)-N(14)-N(18) | -26.8 |
| C(9)-C(12)-N(14)-C(15) | 167.2 | O(13)-C(12)-N(14)-C(15) | -11.9 | N(18)-N(14)-C(15)-C(16) | 0.7 | C(9)-C(12)-N(14)-C(15) | 160.1 | O(13)-C(12)-N(14)-C(15) | -17.6 |
| C(9)-C(12)-N(14)-N(18) | -18.0 | O(13)-C(12)-N(14)-N(18) | 162.5 | N(18)-N(14)-C(15)-C(20) | 179.6 | C(9)-C(12)-N(14)-N(18) | -28.0 | O(13)-C(12)-N(14)-N(18) | 154.0 |
| O(13)-C(12)-N(14)-C(15) | -12.5 | C(9)-C(10)-C(11)-C(6) | -2.3 | C(9)-C(12)-N(14)-C(15) | 168.8 | O(13)-C(12)-N(14)-C(15) | -18.9 | C(9)-C(10)-C(11)-C(6) | -2.2 |
| O(13)-C(12)-N(14)-N(18) | 162.3 | C(8)-C(9)-C(12)-O(13) | -23.5 | C(9)-C(12)-N(14)-N(18) | -16.9 | O(13)-C(12)-N(14)-N(18) | 152.9 | C(8)-C(9)-C(12)-O(13) | -22.8 |
| C(9)-C(10)-C(11)-C(6) | -2.2 | C(8)-C(9)-C(12)-N(14) | 156.9 | O(13)-C(12)-N(14)-C(15) | -11.0 | C(9)-C(10)-C(11)-C(6) | -2.0 | C(8)-C(9)-C(12)-N(14) | 158.0 |
| C(8)-C(9)-C(12)-O(13) | -22.0 | C(10)-C(9)-C(12)-O(13) | 151.2 | O(13)-C(12)-N(14)-N(18) | 163.3 | C(8)-C(9)-C(12)-O(13) | -21.0 | C(10)-C(9)-C(12)-O(13) | 152.0 |
| C(8)-C(9)-C(12)-N(14) | 158.4 | C(10)-C(9)-C(12)-N(14) | -28.4 | C(9)-C(10)-C(11)-C(6) | -2.5 | C(8)-C(9)-C(12)-N(14) | 160.0 | C(10)-C(9)-C(12)-N(14) | -27.2 |
| C(10)-C(9)-C(12)-O(13) | 152.9 | C(8)-C(9)-C(10)-C(11) | 0.2 | C(8)-C(9)-C(12)-O(13) | -24.3 | C(10)-C(9)-C(12)-O(13) | 153.9 | C(8)-C(9)-C(10)-C(11) | 0.3 |
| C(10)-C(9)-C(12)-N(14) | -26.7 | C(12)-C(9)-C(10)-C(11) | -174.4 | C(8)-C(9)-C(12)-N(14) | 155.9 | C(10)-C(9)-C(12)-N(14) | -25.1 | C(12)-C(9)-C(10)-C(11) | -174.4 |
| C(8)-C(9)-C(10)-C(11) | 0.0 | C(7)-C(8)-C(9)-C(10) | 2.3 | C(10)-C(9)-C(12)-O(13) | 150.3 | C(8)-C(9)-C(10)-C(11) | 0.2 | C(7)-C(8)-C(9)-C(10) | 2.0 |
| C(12)-C(9)-C(10)-C(11) | -174.7 | C(7)-C(8)-C(9)-C(12) | 177.4 | C(10)-C(9)-C(12)-N(14) | -29.5 | C(12)-C(9)-C(10)-C(11) | -174.6 | C(7)-C(8)-C(9)-C(12) | 177.1 |
| C(7)-C(8)-C(9)-C(10) | 2.2 | C(6)-C(7)-C(8)-C(9) | -2.6 | C(8)-C(9)-C(10)-C(11) | 0.2 | C(7)-C(8)-C(9)-C(10) | 1.9 | C(6)-C(7)-C(8)-C(9) | -2.4 |
| C(7)-C(8)-C(9)-C(12) | 177.5 | N(5)-C(6)-C(11)-C(10) | 179.0 | C(12)-C(9)-C(10)-C(11) | -174.3 | C(7)-C(8)-C(9)-C(12) | 177.1 | N(5)-C(6)-C(11)-C(10) | 178.7 |
| C(6)-C(7)-C(8)-C(9) | -2.4 | C(7)-C(6)-C(11)-C(10) | 2.1 | C(7)-C(8)-C(9)-C(10) | 2.3 | C(6)-C(7)-C(8)-C(9) | -2.2 | C(7)-C(6)-C(11)-C(10) | 1.9 |
| N(5)-C(6)-C(11)-C(10) | 178.8 | N(5)-C(6)-C(7)-C(8) | -176.6 | C(7)-C(8)-C(9)-C(12) | 177.3 | N(5)-C(6)-C(11)-C(10) | 178.5 | N(5)-C(6)-C(7)-C(8) | -176.4 |
| C(7)-C(6)-C(11)-C(10) | 2.1 | C(11)-C(6)-C(7)-C(8) | 0.3 | C(6)-C(7)-C(8)-C(9) | -2.6 | C(7)-C(6)-C(11)-C(10) | 1.8 | C(11)-C(6)-C(7)-C(8) | 0.4 |
| N(5)-C(6)-C(7)-C(8) | -176.6 | S(2)-N(5)-C(6)-C(7) | -128.5 | N(5)-C(6)-C(11)-C(10) | 179.1 | N(5)-C(6)-C(7)-C(8) | -176.4 | S(2)-N(5)-C(6)-C(7) | -126.0 |
| C(11)-C(6)-C(7)-C(8) | 0.2 | S(2)-N(5)-C(6)-C(11) | 54.6 | C(7)-C(6)-C(11)-C(10) | 2.2 | C(11)-C(6)-C(7)-C(8) | 0.3 | S(2)-N(5)-C(6)-C(11) | 57.2 |
| S(2)-N(5)-C(6)-C(7) | -130.4 | C(1)-S(2)-N(5)-C(6) | 60.5 | N(5)-C(6)-C(7)-C(8) | -176.6 | S(2)-N(5)-C(6)-C(7) | -126.3 | C(1)-S(2)-N(5)-C(6) | 61.6 |
| S(2)-N(5)-C(6)-C(11) | 52.8 | O(3)-S(2)-N(5)-C(6) | 174.2 | C(11)-C(6)-C(7)-C(8) | 0.4 | S(2)-N(5)-C(6)-C(11) | 56.9 | O(3)-S(2)-N(5)-C(6) | 175.3 |
| C(1)-S(2)-N(5)-C(6) | 59.5 | O(4)-S(2)-N(5)-C(6) | -54.4 | S(2)-N(5)-C(6)-C(7) | -128.5 | C(1)-S(2)-N(5)-C(6) | 61.4 | O(4)-S(2)-N(5)-C(6) | -53.3 |
| O(3)-S(2)-N(5)-C(6) | 173.5 | S(2)-C(1)-C(25)-C(24) | 178.8 | S(2)-N(5)-C(6)-C(11) | 54.5 | O(3)-S(2)-N(5)-C(6) | 175.4 | S(2)-C(1)-C(25)-C(24) | 178.8 |
| O(4)-S(2)-N(5)-C(6) | -55.9 | C(21)-C(1)-C(25)-C(24) | -0.9 | C(1)-S(2)-N(5)-C(6) | 60.7 | O(4)-S(2)-N(5)-C(6) | -53.8 | C(21)-C(1)-C(25)-C(24) | -0.8 |
| S(2)-C(1)-C(25)-C(24) | 178.7 | S(2)-C(1)-C(21)-C(22) | -179.1 | O(3)-S(2)-N(5)-C(6) | 174.2 | S(2)-C(1)-C(25)-C(24) | 178.6 | S(2)-C(1)-C(21)-C(22) | -179.0 |
| C(21)-C(1)-C(25)-C(24) | -0.8 | C(25)-C(1)-C(21)-C(22) | 0.6 | O(4)-S(2)-N(5)-C(6) | -53.8 | C(21)-C(1)-C(25)-C(24) | -0.7 | C(25)-C(1)-C(21)-C(22) | 0.6 |
| S(2)-C(1)-C(21)-C(22) | -178.8 | C(21)-C(1)-S(2)-O(3) | -18.5 | S(2)-C(1)-C(25)-C(24) | 178.7 | S(2)-C(1)-C(21)-C(22) | -178.8 | C(21)-C(1)-S(2)-O(3) | -18.5 |
| C(25)-C(1)-C(21)-C(22) | 0.6 | C(21)-C(1)-S(2)-O(4) | -153.4 | C(21)-C(1)-C(25)-C(24) | -1.0 | C(25)-C(1)-C(21)-C(22) | 0.5 | C(21)-C(1)-S(2)-O(4) | -153.4 |
| C(21)-C(1)-S(2)-O(3) | -21.2 | C(21)-C(1)-S(2)-N(5) | 92.1 | S(2)-C(1)-C(21)-C(22) | -178.9 | C(21)-C(1)-S(2)-O(3) | -20.0 | C(21)-C(1)-S(2)-N(5) | 92.2 |
| C(21)-C(1)-S(2)-O(4) | -156.4 | C(25)-C(1)-S(2)-O(3) | 161.8 | C(25)-C(1)-C(21)-C(22) | 0.8 | C(21)-C(1)-S(2)-O(4) | -155.2 | C(25)-C(1)-S(2)-O(3) | 161.9 |
| C(21)-C(1)-S(2)-N(5) | 89.1 | C(25)-C(1)-S(2)-O(4) | 26.9 | C(21)-C(1)-S(2)-O(3) | -19.5 | C(21)-C(1)-S(2)-N(5) | 90.6 | C(25)-C(1)-S(2)-O(4) | 27.0 |
| C(25)-C(1)-S(2)-O(3) | 159.3 | C(25)-C(1)-S(2)-N(5) | -87.6 | C(21)-C(1)-S(2)-O(4) | -154.2 | C(25)-C(1)-S(2)-O(3) | 160.7 | C(25)-C(1)-S(2)-N(5) | -87.4 |
| C(25)-C(1)-S(2)-O(4) | 24.1 |  |  | C(21)-C(1)-S(2)-N(5) | 91.2 | C(25)-C(1)-S(2)-O(4) | 25.5 |  |  |
| C(25)-C(1)-S(2)-N(5) | -90.3 |  |  | C(25)-C(1)-S(2)-O(3) | 160.8 | C(25)-C(1)-S(2)-N(5) | -88.8 |  |  |
|  |  |  |  | C(25)-C(1)-S(2)-O(4) | 26.1 |  |  |  |  |
|  |  |  |  | C(25)-C(1)-S(2)-N(5) | -88.5 |  |  |  |  |
|  | **6c** |  | **7a** |  | **7b** |  | **7c** |  |  |
| C(23)-C(24)-C(25)-C(1) | 0.4 | C(22)-C(23)-C(24)-C(1) | 0.2 | C(22)-C(23)-C(24)-C(1) | 0.4 | C(22)-C(23)-C(24)-C(1) | 0.5 |  |  |
| C(22)-C(23)-N(26)-O(27) | -1.5 | C(21)-C(22)-O(25)-C(26) | 179.9 | C(21)-C(22)-C(23)-C(24) | 0.4 | C(21)-C(22)-N(27)-O(28) | -1.8 |  |  |
| C(22)-C(23)-N(26)-O(28) | 179.6 | C(23)-C(22)-O(25)-C(26) | 0.3 | Cl(25)-C(22)-C(23)-C(24) | 179.9 | C(21)-C(22)-N(27)-O(29) | -179.9 |  |  |
| C(24)-C(23)-N(26)-O(27) | 179.2 | C(21)-C(22)-C(23)-C(24) | 0.5 | C(20)-C(21)-C(22)-C(23) | -0.6 | C(23)-C(22)-N(27)-O(28) | 179.1 |  |  |
| C(24)-C(23)-N(26)-O(28) | 0.3 | O(25)-C(22)-C(23)-C(24) | -179.9 | C(20)-C(21)-C(22)-Cl(25) | 179.9 | C(23)-C(22)-N(27)-O(29) | 0.9 |  |  |
| C(22)-C(23)-C(24)-C(25) | 0.4 | C(20)-C(21)-C(22)-C(23) | -0.6 | C(1)-C(20)-C(21)-C(22) | 0.0 | C(21)-C(22)-C(23)-C(24) | 0.4 |  |  |
| N(26)-C(23)-C(24)-C(25) | 179.6 | C(20)-C(21)-C(22)-O(25) | 179.8 | C(16)-C(17)-N(18)-N(14) | 0.3 | N(27)-C(22)-C(23)-C(24) | 179.5 |  |  |
| C(21)-C(22)-C(23)-C(24) | -0.5 | C(1)-C(20)-C(21)-C(22) | -0.1 | C(15)-C(16)-C(26)-N(27) | 4.2 | C(20)-C(21)-C(22)-C(23) | -0.6 |  |  |
| C(21)-C(22)-C(23)-N(26) | -179.9 | C(16)-C(17)-N(18)-N(14) | 0.3 | C(17)-C(16)-C(26)-N(27) | -174.9 | C(20)-C(21)-C(22)-N(27) | -179.7 |  |  |
| C(1)-C(21)-C(22)-C(23) | 0.0 | C(15)-C(16)-C(27)-N(28) | 5.9 | C(15)-C(16)-C(17)-N(18) | 0.2 | C(1)-C(20)-C(21)-C(22) | 0.0 |  |  |
| C(16)-C(17)-N(18)-N(14) | -0.2 | C(17)-C(16)-C(27)-N(28) | -173.1 | C(26)-C(16)-C(17)-N(18) | 179.4 | C(16)-C(17)-N(18)-N(14) | 0.3 |  |  |
| C(19)-C(17)-N(18)-N(14) | 179.3 | C(15)-C(16)-C(17)-N(18) | 0.1 | N(14)-C(15)-C(16)-C(17) | -0.6 | C(15)-C(16)-C(25)-N(26) | 3.1 |  |  |
| C(15)-C(16)-C(17)-N(18) | 0.1 | C(27)-C(16)-C(17)-N(18) | 179.3 | N(14)-C(15)-C(16)-C(26) | -179.8 | C(17)-C(16)-C(25)-N(26) | -176.0 |  |  |
| C(15)-C(16)-C(17)-C(19) | -179.4 | N(14)-C(15)-C(16)-C(17) | -0.5 | N(19)-C(15)-C(16)-C(17) | -177.3 | C(15)-C(16)-C(17)-N(18) | 0.2 |  |  |
| N(14)-C(15)-C(16)-C(17) | 0.0 | N(14)-C(15)-C(16)-C(27) | -179.7 | N(19)-C(15)-C(16)-C(26) | 3.5 | C(25)-C(16)-C(17)-N(18) | 179.4 |  |  |
| O(20)-C(15)-C(16)-C(17) | -178.1 | N(19)-C(15)-C(16)-C(17) | -177.2 | C(12)-N(14)-N(18)-C(17) | -175.4 | N(14)-C(15)-C(16)-C(17) | -0.6 |  |  |
| C(12)-N(14)-N(18)-C(17) | -172.4 | N(19)-C(15)-C(16)-C(27) | 3.6 | C(15)-N(14)-N(18)-C(17) | -0.7 | N(14)-C(15)-C(16)-C(25) | -179.9 |  |  |
| C(15)-N(14)-N(18)-C(17) | 0.2 | C(12)-N(14)-N(18)-C(17) | -175.4 | C(12)-N(14)-C(15)-C(16) | 175.4 | N(19)-C(15)-C(16)-C(17) | -177.2 |  |  |
| C(12)-N(14)-C(15)-C(16) | 172.0 | C(15)-N(14)-N(18)-C(17) | -0.7 | C(12)-N(14)-C(15)-N(19) | -7.7 | N(19)-C(15)-C(16)-C(25) | 3.5 |  |  |
| C(12)-N(14)-C(15)-O(20) | -9.9 | C(12)-N(14)-C(15)-C(16) | 175.4 | N(18)-N(14)-C(15)-C(16) | 0.8 | C(12)-N(14)-N(18)-C(17) | -175.4 |  |  |
| N(18)-N(14)-C(15)-C(16) | -0.1 | C(12)-N(14)-C(15)-N(19) | -7.7 | N(18)-N(14)-C(15)-N(19) | 177.7 | C(15)-N(14)-N(18)-C(17) | -0.7 |  |  |
| N(18)-N(14)-C(15)-O(20) | 178.0 | N(18)-N(14)-C(15)-C(16) | 0.8 | C(9)-C(12)-N(14)-C(15) | 172.6 | C(12)-N(14)-C(15)-C(16) | 175.4 |  |  |
| C(9)-C(12)-N(14)-C(15) | 162.8 | N(18)-N(14)-C(15)-N(19) | 177.7 | C(9)-C(12)-N(14)-N(18) | -13.4 | C(12)-N(14)-C(15)-N(19) | -7.7 |  |  |
| C(9)-C(12)-N(14)-N(18) | -25.7 | C(9)-C(12)-N(14)-C(15) | 172.6 | O(13)-C(12)-N(14)-C(15) | -7.4 | N(18)-N(14)-C(15)-C(16) | 0.8 |  |  |
| O(13)-C(12)-N(14)-C(15) | -16.5 | C(9)-C(12)-N(14)-N(18) | -13.5 | O(13)-C(12)-N(14)-N(18) | 166.6 | N(18)-N(14)-C(15)-N(19) | 177.7 |  |  |
| O(13)-C(12)-N(14)-N(18) | 155.0 | O(13)-C(12)-N(14)-C(15) | -7.5 | C(9)-C(10)-C(11)-C(6) | -2.2 | C(9)-C(12)-N(14)-C(15) | 172.7 |  |  |
| C(9)-C(10)-C(11)-C(6) | -2.4 | O(13)-C(12)-N(14)-N(18) | 166.5 | C(8)-C(9)-C(12)-O(13) | -19.8 | C(9)-C(12)-N(14)-N(18) | -13.3 |  |  |
| C(8)-C(9)-C(12)-O(13) | -23.8 | C(9)-C(10)-C(11)-C(6) | -2.1 | C(8)-C(9)-C(12)-N(14) | 160.2 | O(13)-C(12)-N(14)-C(15) | -7.3 |  |  |
| C(8)-C(9)-C(12)-N(14) | 156.9 | C(8)-C(9)-C(12)-O(13) | -19.0 | C(10)-C(9)-C(12)-O(13) | 155.5 | O(13)-C(12)-N(14)-N(18) | 166.7 |  |  |
| C(10)-C(9)-C(12)-O(13) | 150.9 | C(8)-C(9)-C(12)-N(14) | 161.0 | C(10)-C(9)-C(12)-N(14) | -24.5 | C(9)-C(10)-C(11)-C(6) | -2.3 |  |  |
| C(10)-C(9)-C(12)-N(14) | -28.4 | C(10)-C(9)-C(12)-O(13) | 156.5 | C(8)-C(9)-C(10)-C(11) | 0.1 | C(8)-C(9)-C(12)-O(13) | -21.2 |  |  |
| C(8)-C(9)-C(10)-C(11) | 0.3 | C(10)-C(9)-C(12)-N(14) | -23.6 | C(12)-C(9)-C(10)-C(11) | -175.1 | C(8)-C(9)-C(12)-N(14) | 158.8 |  |  |
| C(12)-C(9)-C(10)-C(11) | -174.2 | C(8)-C(9)-C(10)-C(11) | 0.0 | C(7)-C(8)-C(9)-C(10) | 2.2 | C(10)-C(9)-C(12)-O(13) | 154.0 |  |  |
| C(7)-C(8)-C(9)-C(10) | 2.1 | C(12)-C(9)-C(10)-C(11) | -175.4 | C(7)-C(8)-C(9)-C(12) | 177.8 | C(10)-C(9)-C(12)-N(14) | -26.0 |  |  |
| C(7)-C(8)-C(9)-C(12) | 177.1 | C(7)-C(8)-C(9)-C(10) | 2.1 | C(6)-C(7)-C(8)-C(9) | -2.3 | C(8)-C(9)-C(10)-C(11) | 0.2 |  |  |
| C(6)-C(7)-C(8)-C(9) | -2.5 | C(7)-C(8)-C(9)-C(12) | 178.0 | N(5)-C(6)-C(11)-C(10) | 178.8 | C(12)-C(9)-C(10)-C(11) | -174.9 |  |  |
| N(5)-C(6)-C(11)-C(10) | 178.8 | C(6)-C(7)-C(8)-C(9) | -2.2 | C(7)-C(6)-C(11)-C(10) | 2.0 | C(7)-C(8)-C(9)-C(10) | 2.2 |  |  |
| C(7)-C(6)-C(11)-C(10) | 2.0 | N(5)-C(6)-C(11)-C(10) | 179.0 | N(5)-C(6)-C(7)-C(8) | -176.7 | C(7)-C(8)-C(9)-C(12) | 177.7 |  |  |
| N(5)-C(6)-C(7)-C(8) | -176.5 | C(7)-C(6)-C(11)-C(10) | 2.1 | C(11)-C(6)-C(7)-C(8) | 0.2 | C(6)-C(7)-C(8)-C(9) | -2.5 |  |  |
| C(11)-C(6)-C(7)-C(8) | 0.4 | N(5)-C(6)-C(7)-C(8) | -177.0 | S(2)-N(5)-C(6)-C(7) | -136.1 | N(5)-C(6)-C(11)-C(10) | 178.9 |  |  |
| S(2)-N(5)-C(6)-C(7) | -127.3 | C(11)-C(6)-C(7)-C(8) | 0.1 | S(2)-N(5)-C(6)-C(11) | 47.1 | C(7)-C(6)-C(11)-C(10) | 2.1 |  |  |
| S(2)-N(5)-C(6)-C(11) | 55.9 | S(2)-N(5)-C(6)-C(7) | -135.7 | C(1)-S(2)-N(5)-C(6) | 61.1 | N(5)-C(6)-C(7)-C(8) | -176.6 |  |  |
| C(1)-S(2)-N(5)-C(6) | 61.3 | S(2)-N(5)-C(6)-C(11) | 47.4 | O(3)-S(2)-N(5)-C(6) | 174.8 | C(11)-C(6)-C(7)-C(8) | 0.3 |  |  |
| O(3)-S(2)-N(5)-C(6) | 174.8 | C(1)-S(2)-N(5)-C(6) | 59.4 | O(4)-S(2)-N(5)-C(6) | -53.9 | S(2)-N(5)-C(6)-C(7) | -132.5 |  |  |
| O(4)-S(2)-N(5)-C(6) | -53.1 | O(3)-S(2)-N(5)-C(6) | 173.4 | S(2)-C(1)-C(24)-C(23) | 179.1 | S(2)-N(5)-C(6)-C(11) | 50.6 |  |  |
| S(2)-C(1)-C(25)-C(24) | 178.7 | O(4)-S(2)-N(5)-C(6) | -56.0 | C(20)-C(1)-C(24)-C(23) | -1.0 | C(1)-S(2)-N(5)-C(6) | 62.1 |  |  |
| C(21)-C(1)-C(25)-C(24) | -1.0 | S(2)-C(1)-C(24)-C(23) | 178.7 | S(2)-C(1)-C(20)-C(21) | -179.2 | O(3)-S(2)-N(5)-C(6) | 175.4 |  |  |
| S(2)-C(1)-C(21)-C(22) | -178.9 | C(20)-C(1)-C(24)-C(23) | -0.9 | C(24)-C(1)-C(20)-C(21) | 0.8 | O(4)-S(2)-N(5)-C(6) | -52.5 |  |  |
| C(25)-C(1)-C(21)-C(22) | 0.8 | S(2)-C(1)-C(20)-C(21) | -178.8 | C(20)-C(1)-S(2)-O(3) | -21.3 | S(2)-C(1)-C(24)-C(23) | 178.8 |  |  |
| C(21)-C(1)-S(2)-O(3) | -19.9 | C(24)-C(1)-C(20)-C(21) | 0.8 | C(20)-C(1)-S(2)-O(4) | -156.5 | C(20)-C(1)-C(24)-C(23) | -1.1 |  |  |
| C(21)-C(1)-S(2)-O(4) | -154.6 | C(20)-C(1)-S(2)-O(3) | -24.0 | C(20)-C(1)-S(2)-N(5) | 88.9 | S(2)-C(1)-C(20)-C(21) | -179.0 |  |  |
| C(21)-C(1)-S(2)-N(5) | 90.9 | C(20)-C(1)-S(2)-O(4) | -159.6 | C(24)-C(1)-S(2)-O(3) | 158.7 | C(24)-C(1)-C(20)-C(21) | 0.9 |  |  |
| C(25)-C(1)-S(2)-O(3) | 160.4 | C(20)-C(1)-S(2)-N(5) | 85.9 | C(24)-C(1)-S(2)-O(4) | 23.5 | C(20)-C(1)-S(2)-O(3) | -20.2 |  |  |
| C(25)-C(1)-S(2)-O(4) | 25.7 | C(24)-C(1)-S(2)-O(3) | 156.4 | C(24)-C(1)-S(2)-N(5) | -91.1 | C(20)-C(1)-S(2)-O(4) | -155.2 |  |  |
| C(25)-C(1)-S(2)-N(5) | -88.8 | C(24)-C(1)-S(2)-O(4) | 20.8 |  |  | C(20)-C(1)-S(2)-N(5) | 90.2 |  |  |
|  |  | C(24)-C(1)-S(2)-N(5) | -93.6 |  |  | C(24)-C(1)-S(2)-O(3) | 159.9 |  |  |
|  |  |  |  |  |  | C(24)-C(1)-S(2)-O(4) | 24.9 |  |  |
|  |  |  |  |  |  | C(24)-C(1)-S(2)-N(5) | -89.6 |  |  |

**Table S2**. The DFT bond length data of the investigated compounds.

|  | **5a** |  | **5b** |  | **5c** |  | **6a** |  | **6b** |
| --- | --- | --- | --- | --- | --- | --- | --- | --- | --- |
| O(26)-C(27) | 1.43 | C(24)-C(25) | 1.40 | N(26)-O(28) | 1.30 | O(26)-C(27) | 1.43 | C(24)-C(25) | 1.40 |
| C(24)-C(25) | 1.40 | C(23)-Cl(26) | 1.75 | N(26)-O(27) | 1.13 | C(24)-C(25) | 1.40 | C(23)-Cl(26) | 1.75 |
| C(23)-O(26) | 1.36 | C(23)-C(24) | 1.40 | C(24)-C(25) | 1.40 | C(23)-O(26) | 1.36 | C(23)-C(24) | 1.40 |
| C(23)-C(24) | 1.41 | C(22)-C(23) | 1.40 | C(23)-N(26) | 1.44 | C(23)-C(24) | 1.41 | C(22)-C(23) | 1.40 |
| C(22)-C(23) | 1.41 | C(21)-C(22) | 1.40 | C(23)-C(24) | 1.40 | C(22)-C(23) | 1.41 | C(21)-C(22) | 1.40 |
| C(21)-C(22) | 1.39 | C(17)-C(19) | 1.50 | C(22)-C(23) | 1.40 | C(21)-C(22) | 1.39 | C(17)-C(19) | 1.50 |
| C(17)-C(19) | 1.50 | C(17)-N(18) | 1.32 | C(21)-C(22) | 1.40 | C(17)-C(19) | 1.50 | C(17)-N(18) | 1.29 |
| C(17)-N(18) | 1.32 | C(16)-C(17) | 1.43 | C(17)-C(19) | 1.50 | C(17)-N(18) | 1.29 | C(16)-C(17) | 1.50 |
| C(16)-C(17) | 1.43 | C(15)-C(20) | 1.50 | C(17)-N(18) | 1.32 | C(16)-C(17) | 1.50 | C(15)-O(20) | 1.21 |
| C(15)-C(20) | 1.50 | C(15)-C(16) | 1.38 | C(16)-C(17) | 1.43 | C(15)-O(20) | 1.21 | C(15)-C(16) | 1.53 |
| C(15)-C(16) | 1.38 | N(14)-N(18) | 1.38 | C(15)-C(20) | 1.50 | C(15)-C(16) | 1.53 | N(14)-N(18) | 1.41 |
| N(14)-N(18) | 1.38 | N(14)-C(15) | 1.39 | C(15)-C(16) | 1.37 | N(14)-N(18) | 1.41 | N(14)-C(15) | 1.42 |
| N(14)-C(15) | 1.39 | C(12)-N(14) | 1.42 | N(14)-N(18) | 1.38 | N(14)-C(15) | 1.42 | C(12)-N(14) | 1.42 |
| C(12)-N(14) | 1.42 | C(12)-O(13) | 1.23 | N(14)-C(15) | 1.39 | C(12)-N(14) | 1.42 | C(12)-O(13) | 1.22 |
| C(12)-O(13) | 1.23 | C(10)-C(11) | 1.39 | C(12)-N(14) | 1.41 | C(12)-O(13) | 1.22 | C(10)-C(11) | 1.39 |
| C(10)-C(11) | 1.39 | C(9)-C(12) | 1.49 | C(12)-O(13) | 1.23 | C(10)-C(11) | 1.39 | C(9)-C(12) | 1.50 |
| C(9)-C(12) | 1.49 | C(9)-C(10) | 1.41 | C(10)-C(11) | 1.39 | C(9)-C(12) | 1.50 | C(9)-C(10) | 1.41 |
| C(9)-C(10) | 1.41 | C(8)-C(9) | 1.41 | C(9)-C(12) | 1.50 | C(9)-C(10) | 1.41 | C(8)-C(9) | 1.41 |
| C(8)-C(9) | 1.41 | C(7)-C(8) | 1.39 | C(9)-C(10) | 1.41 | C(8)-C(9) | 1.41 | C(7)-C(8) | 1.39 |
| C(7)-C(8) | 1.39 | C(6)-C(11) | 1.40 | C(8)-C(9) | 1.41 | C(7)-C(8) | 1.39 | C(6)-C(11) | 1.41 |
| C(6)-C(11) | 1.41 | C(6)-C(7) | 1.41 | C(7)-C(8) | 1.39 | C(6)-C(11) | 1.41 | C(6)-C(7) | 1.41 |
| C(6)-C(7) | 1.41 | N(5)-C(6) | 1.42 | C(6)-C(11) | 1.40 | C(6)-C(7) | 1.41 | N(5)-C(6) | 1.42 |
| N(5)-C(6) | 1.42 | S(2)-N(5) | 1.71 | C(6)-C(7) | 1.41 | N(5)-C(6) | 1.42 | S(2)-N(5) | 1.71 |
| S(2)-N(5) | 1.72 | S(2)-O(4) | 1.46 | N(5)-C(6) | 1.42 | S(2)-N(5) | 1.72 | S(2)-O(4) | 1.46 |
| S(2)-O(4) | 1.46 | S(2)-O(3) | 1.46 | S(2)-N(5) | 1.70 | S(2)-O(4) | 1.46 | S(2)-O(3) | 1.46 |
| S(2)-O(3) | 1.46 | C(1)-C(25) | 1.40 | S(2)-O(4) | 1.46 | S(2)-O(3) | 1.46 | C(1)-C(25) | 1.40 |
| C(1)-C(25) | 1.40 | C(1)-C(21) | 1.40 | S(2)-O(3) | 1.46 | C(1)-C(25) | 1.40 | C(1)-C(21) | 1.40 |
| C(1)-C(21) | 1.40 | C(1)-S(2) | 1.80 | C(1)-C(25) | 1.40 | C(1)-C(21) | 1.40 | C(1)-S(2) | 1.80 |
| C(1)-S(2) | 1.79 |  |  | C(1)-C(21) | 1.40 | C(1)-S(2) | 1.79 |  |  |
|  |  |  |  | C(1)-S(2) | 1.81 |  |  |  |  |
|  | **6c** |  | **7a** |  | **7b** |  | **7c** |  |  |
| N(26)-O(28) | 1.30 | C(27)-N(28) | 1.2 | C(26)-N(27) | 1.17 | N(27)-O(29) | 1.30 |  |  |
| N(26)-O(27) | 1.13 | O(25)-C(26) | 1.4 | C(23)-C(24) | 1.40 | N(27)-O(28) | 1.13 |  |  |
| C(24)-C(25) | 1.40 | C(23)-C(24) | 1.4 | C(22)-Cl(25) | 1.75 | C(25)-N(26) | 1.17 |  |  |
| C(23)-N(26) | 1.44 | C(22)-O(25) | 1.4 | C(22)-C(23) | 1.40 | C(23)-C(24) | 1.40 |  |  |
| C(23)-C(24) | 1.40 | C(22)-C(23) | 1.4 | C(21)-C(22) | 1.40 | C(22)-N(27) | 1.44 |  |  |
| C(22)-C(23) | 1.40 | C(21)-C(22) | 1.4 | C(20)-C(21) | 1.40 | C(22)-C(23) | 1.40 |  |  |
| C(21)-C(22) | 1.40 | C(20)-C(21) | 1.4 | C(17)-N(18) | 1.31 | C(21)-C(22) | 1.40 |  |  |
| C(17)-C(19) | 1.50 | C(17)-N(18) | 1.3 | C(16)-C(26) | 1.42 | C(20)-C(21) | 1.40 |  |  |
| C(17)-N(18) | 1.29 | C(16)-C(27) | 1.4 | C(16)-C(17) | 1.43 | C(17)-N(18) | 1.31 |  |  |
| C(16)-C(17) | 1.50 | C(16)-C(17) | 1.4 | C(15)-N(19) | 1.36 | C(16)-C(25) | 1.42 |  |  |
| C(15)-O(20) | 1.21 | C(15)-N(19) | 1.4 | C(15)-C(16) | 1.40 | C(16)-C(17) | 1.43 |  |  |
| C(15)-C(16) | 1.53 | C(15)-C(16) | 1.4 | N(14)-N(18) | 1.40 | C(15)-N(19) | 1.36 |  |  |
| N(14)-N(18) | 1.41 | N(14)-N(18) | 1.4 | N(14)-C(15) | 1.39 | C(15)-C(16) | 1.40 |  |  |
| N(14)-C(15) | 1.42 | N(14)-C(15) | 1.4 | C(12)-N(14) | 1.41 | N(14)-N(18) | 1.40 |  |  |
| C(12)-N(14) | 1.42 | C(12)-N(14) | 1.4 | C(12)-O(13) | 1.23 | N(14)-C(15) | 1.39 |  |  |
| C(12)-O(13) | 1.22 | C(12)-O(13) | 1.2 | C(10)-C(11) | 1.39 | C(12)-N(14) | 1.41 |  |  |
| C(10)-C(11) | 1.39 | C(10)-C(11) | 1.4 | C(9)-C(12) | 1.49 | C(12)-O(13) | 1.23 |  |  |
| C(9)-C(12) | 1.50 | C(9)-C(12) | 1.5 | C(9)-C(10) | 1.41 | C(10)-C(11) | 1.39 |  |  |
| C(9)-C(10) | 1.41 | C(9)-C(10) | 1.4 | C(8)-C(9) | 1.41 | C(9)-C(12) | 1.49 |  |  |
| C(8)-C(9) | 1.40 | C(8)-C(9) | 1.4 | C(7)-C(8) | 1.39 | C(9)-C(10) | 1.41 |  |  |
| C(7)-C(8) | 1.39 | C(7)-C(8) | 1.4 | C(6)-C(11) | 1.41 | C(8)-C(9) | 1.41 |  |  |
| C(6)-C(11) | 1.40 | C(6)-C(11) | 1.4 | C(6)-C(7) | 1.41 | C(7)-C(8) | 1.39 |  |  |
| C(6)-C(7) | 1.41 | C(6)-C(7) | 1.4 | N(5)-C(6) | 1.42 | C(6)-C(11) | 1.41 |  |  |
| N(5)-C(6) | 1.42 | N(5)-C(6) | 1.4 | S(2)-N(5) | 1.71 | C(6)-C(7) | 1.41 |  |  |
| S(2)-N(5) | 1.70 | S(2)-N(5) | 1.7 | S(2)-O(4) | 1.46 | N(5)-C(6) | 1.42 |  |  |
| S(2)-O(4) | 1.46 | S(2)-O(4) | 1.5 | S(2)-O(3) | 1.46 | S(2)-N(5) | 1.70 |  |  |
| S(2)-O(3) | 1.46 | S(2)-O(3) | 1.5 | C(1)-C(24) | 1.40 | S(2)-O(4) | 1.46 |  |  |
| C(1)-C(25) | 1.40 | C(1)-C(24) | 1.4 | C(1)-C(20) | 1.40 | S(2)-O(3) | 1.46 |  |  |
| C(1)-C(21) | 1.40 | C(1)-C(20) | 1.4 | C(1)-S(2) | 1.80 | C(1)-C(24) | 1.40 |  |  |
| C(1)-S(2) | 1.81 | C(1)-S(2) | 1.8 |  |  | C(1)-C(20) | 1.40 |  |  |
|  |  |  |  |  |  | C(1)-S(2) | 1.81 |  |  |

**Table S3.** The DFT bond angle data of the investigated compounds.

|  | **5a** |  | **5b** |  | **5c** |  | **6a** |  | **6b** |
| --- | --- | --- | --- | --- | --- | --- | --- | --- | --- |
| C(27)-O(26)-C(23) | 118.6 | C(24)-C(25)-C(1) | 119.3 | O(28)-N(26)-O(27) | 122.1 | C(27)-O(26)-C(23) | 118.6 | C(24)-C(25)-C(1) | 119.3 |
| C(24)-C(25)-C(1) | 119.9 | C(25)-C(24)-C(23) | 119.4 | O(28)-N(26)-C(23) | 115.8 | C(24)-C(25)-C(1) | 119.9 | C(25)-C(24)-C(23) | 119.4 |
| C(25)-C(24)-C(23) | 119.7 | Cl(26)-C(23)-C(24) | 119.4 | O(27)-N(26)-C(23) | 122.1 | C(25)-C(24)-C(23) | 119.7 | Cl(26)-C(23)-C(24) | 119.4 |
| O(26)-C(23)-C(24) | 124.5 | Cl(26)-C(23)-C(22) | 119.3 | C(24)-C(25)-C(1) | 119.1 | O(26)-C(23)-C(24) | 124.5 | Cl(26)-C(23)-C(22) | 119.3 |
| O(26)-C(23)-C(22) | 115.5 | C(24)-C(23)-C(22) | 121.3 | C(25)-C(24)-C(23) | 118.8 | O(26)-C(23)-C(22) | 115.5 | C(24)-C(23)-C(22) | 121.3 |
| C(24)-C(23)-C(22) | 119.9 | C(23)-C(22)-C(21) | 119.3 | N(26)-C(23)-C(24) | 121.0 | C(24)-C(23)-C(22) | 119.9 | C(23)-C(22)-C(21) | 119.3 |
| C(23)-C(22)-C(21) | 120.2 | C(22)-C(21)-C(1) | 119.4 | N(26)-C(23)-C(22) | 116.6 | C(23)-C(22)-C(21) | 120.2 | C(22)-C(21)-C(1) | 119.4 |
| C(22)-C(21)-C(1) | 119.5 | C(17)-N(18)-N(14) | 105.4 | C(24)-C(23)-C(22) | 122.4 | C(22)-C(21)-C(1) | 119.5 | C(17)-N(18)-N(14) | 108.7 |
| C(17)-N(18)-N(14) | 105.4 | C(19)-C(17)-N(18) | 120.9 | C(23)-C(22)-C(21) | 118.7 | C(17)-N(18)-N(14) | 108.7 | C(19)-C(17)-N(18) | 121.6 |
| C(19)-C(17)-N(18) | 120.9 | C(19)-C(17)-C(16) | 128.0 | C(22)-C(21)-C(1) | 119.2 | C(19)-C(17)-N(18) | 121.6 | C(19)-C(17)-C(16) | 125.5 |
| C(19)-C(17)-C(16) | 128.1 | N(18)-C(17)-C(16) | 111.1 | C(17)-N(18)-N(14) | 105.3 | C(19)-C(17)-C(16) | 125.5 | N(18)-C(17)-C(16) | 112.9 |
| N(18)-C(17)-C(16) | 111.1 | C(17)-C(16)-C(15) | 106.4 | C(19)-C(17)-N(18) | 120.9 | N(18)-C(17)-C(16) | 112.9 | C(17)-C(16)-C(15) | 102.3 |
| C(17)-C(16)-C(15) | 106.4 | C(20)-C(15)-C(16) | 129.0 | C(19)-C(17)-C(16) | 128.0 | C(17)-C(16)-C(15) | 102.3 | O(20)-C(15)-C(16) | 128.3 |
| C(20)-C(15)-C(16) | 128.9 | C(20)-C(15)-N(14) | 125.5 | N(18)-C(17)-C(16) | 111.1 | O(20)-C(15)-C(16) | 128.2 | O(20)-C(15)-N(14) | 127.7 |
| C(20)-C(15)-N(14) | 125.6 | C(16)-C(15)-N(14) | 105.5 | C(17)-C(16)-C(15) | 106.4 | O(20)-C(15)-N(14) | 127.8 | C(16)-C(15)-N(14) | 104.0 |
| C(16)-C(15)-N(14) | 105.5 | N(18)-N(14)-C(15) | 111.7 | C(20)-C(15)-C(16) | 129.0 | C(16)-C(15)-N(14) | 104.0 | N(18)-N(14)-C(15) | 112.2 |
| N(18)-N(14)-C(15) | 111.6 | N(18)-N(14)-C(12) | 121.6 | C(20)-C(15)-N(14) | 125.5 | N(18)-N(14)-C(15) | 112.2 | N(18)-N(14)-C(12) | 121.3 |
| N(18)-N(14)-C(12) | 121.7 | C(15)-N(14)-C(12) | 126.6 | C(16)-C(15)-N(14) | 105.5 | N(18)-N(14)-C(12) | 121.4 | C(15)-N(14)-C(12) | 126.0 |
| C(15)-N(14)-C(12) | 126.5 | N(14)-C(12)-O(13) | 119.2 | N(18)-N(14)-C(15) | 111.7 | C(15)-N(14)-C(12) | 126.0 | N(14)-C(12)-O(13) | 119.6 |
| N(14)-C(12)-O(13) | 118.9 | N(14)-C(12)-C(9) | 119.4 | N(18)-N(14)-C(12) | 121.5 | N(14)-C(12)-O(13) | 119.4 | N(14)-C(12)-C(9) | 118.4 |
| N(14)-C(12)-C(9) | 119.6 | O(13)-C(12)-C(9) | 121.4 | C(15)-N(14)-C(12) | 126.6 | N(14)-C(12)-C(9) | 118.5 | O(13)-C(12)-C(9) | 122.0 |
| O(13)-C(12)-C(9) | 121.5 | C(10)-C(11)-C(6) | 120.0 | N(14)-C(12)-O(13) | 119.4 | O(13)-C(12)-C(9) | 122.2 | C(10)-C(11)-C(6) | 119.9 |
| C(10)-C(11)-C(6) | 120.1 | C(11)-C(10)-C(9) | 120.9 | N(14)-C(12)-C(9) | 119.4 | C(10)-C(11)-C(6) | 119.9 | C(11)-C(10)-C(9) | 120.9 |
| C(11)-C(10)-C(9) | 120.9 | C(12)-C(9)-C(10) | 125.1 | O(13)-C(12)-C(9) | 121.2 | C(11)-C(10)-C(9) | 121.0 | C(12)-C(9)-C(10) | 124.6 |
| C(12)-C(9)-C(10) | 125.4 | C(12)-C(9)-C(8) | 116.0 | C(10)-C(11)-C(6) | 119.9 | C(12)-C(9)-C(10) | 124.7 | C(12)-C(9)-C(8) | 116.5 |
| C(12)-C(9)-C(8) | 115.9 | C(10)-C(9)-C(8) | 118.7 | C(11)-C(10)-C(9) | 120.8 | C(12)-C(9)-C(8) | 116.5 | C(10)-C(9)-C(8) | 118.7 |
| C(10)-C(9)-C(8) | 118.5 | C(9)-C(8)-C(7) | 120.8 | C(12)-C(9)-C(10) | 125.1 | C(10)-C(9)-C(8) | 118.7 | C(9)-C(8)-C(7) | 120.7 |
| C(9)-C(8)-C(7) | 120.9 | C(8)-C(7)-C(6) | 120.1 | C(12)-C(9)-C(8) | 115.9 | C(9)-C(8)-C(7) | 120.8 | C(8)-C(7)-C(6) | 120.1 |
| C(8)-C(7)-C(6) | 120.2 | C(11)-C(6)-C(7) | 119.5 | C(10)-C(9)-C(8) | 118.7 | C(8)-C(7)-C(6) | 120.2 | C(11)-C(6)-C(7) | 119.5 |
| C(11)-C(6)-C(7) | 119.3 | C(11)-C(6)-N(5) | 121.1 | C(9)-C(8)-C(7) | 120.8 | C(11)-C(6)-C(7) | 119.4 | C(11)-C(6)-N(5) | 120.9 |
| C(11)-C(6)-N(5) | 121.3 | C(7)-C(6)-N(5) | 119.3 | C(8)-C(7)-C(6) | 120.1 | C(11)-C(6)-N(5) | 120.9 | C(7)-C(6)-N(5) | 119.5 |
| C(7)-C(6)-N(5) | 119.3 | C(6)-N(5)-S(2) | 123.5 | C(11)-C(6)-C(7) | 119.6 | C(7)-C(6)-N(5) | 119.6 | C(6)-N(5)-S(2) | 123.2 |
| C(6)-N(5)-S(2) | 123.5 | N(5)-S(2)-O(4) | 107.1 | C(11)-C(6)-N(5) | 121.1 | C(6)-N(5)-S(2) | 122.9 | N(5)-S(2)-O(4) | 107.0 |
| N(5)-S(2)-O(4) | 106.8 | N(5)-S(2)-O(3) | 103.6 | C(7)-C(6)-N(5) | 119.2 | N(5)-S(2)-O(4) | 106.6 | N(5)-S(2)-O(3) | 103.7 |
| N(5)-S(2)-O(3) | 103.1 | N(5)-S(2)-C(1) | 106.2 | C(6)-N(5)-S(2) | 123.8 | N(5)-S(2)-O(3) | 103.3 | N(5)-S(2)-C(1) | 106.2 |
| N(5)-S(2)-C(1) | 106.5 | O(4)-S(2)-O(3) | 123.0 | N(5)-S(2)-O(4) | 107.5 | N(5)-S(2)-C(1) | 106.4 | O(4)-S(2)-O(3) | 123.1 |
| O(4)-S(2)-O(3) | 122.8 | O(4)-S(2)-C(1) | 107.8 | N(5)-S(2)-O(3) | 103.9 | O(4)-S(2)-O(3) | 122.9 | O(4)-S(2)-C(1) | 107.7 |
| O(4)-S(2)-C(1) | 108.1 | O(3)-S(2)-C(1) | 108.0 | N(5)-S(2)-C(1) | 105.9 | O(4)-S(2)-C(1) | 108.2 | O(3)-S(2)-C(1) | 108.0 |
| O(3)-S(2)-C(1) | 108.4 | C(25)-C(1)-C(21) | 121.3 | O(4)-S(2)-O(3) | 123.2 | O(3)-S(2)-C(1) | 108.3 | C(25)-C(1)-C(21) | 121.3 |
| C(25)-C(1)-C(21) | 120.7 | C(25)-C(1)-S(2) | 119.7 | O(4)-S(2)-C(1) | 107.4 | C(25)-C(1)-C(21) | 120.7 | C(25)-C(1)-S(2) | 119.7 |
| C(25)-C(1)-S(2) | 119.9 | C(21)-C(1)-S(2) | 119.1 | O(3)-S(2)-C(1) | 107.8 | C(25)-C(1)-S(2) | 119.9 | C(21)-C(1)-S(2) | 119.1 |
| C(21)-C(1)-S(2) | 119.4 |  |  | C(25)-C(1)-C(21) | 121.8 | C(21)-C(1)-S(2) | 119.4 |  |  |
|  |  |  |  | C(25)-C(1)-S(2) | 119.4 |  |  |  |  |
|  |  |  |  | C(21)-C(1)-S(2) | 118.8 |  |  |  |  |
|  | **6c** |  | **7a** |  | **7b** |  | **7c** |  |  |
| O(28)-N(26)-O(27) | 122.1 | N(28)-C(27)-C(16) | 178.3 | N(27)-C(26)-C(16) | 178.3 | O(29)-N(27)-O(28) | 122.1 |  |  |
| O(28)-N(26)-C(23) | 115.8 | C(26)-O(25)-C(22) | 118.7 | C(23)-C(24)-C(1) | 119.3 | O(29)-N(27)-C(22) | 115.8 |  |  |
| O(27)-N(26)-C(23) | 122.1 | C(23)-C(24)-C(1) | 119.8 | C(24)-C(23)-C(22) | 119.4 | O(28)-N(27)-C(22) | 122.1 |  |  |
| C(24)-C(25)-C(1) | 119.1 | C(24)-C(23)-C(22) | 119.7 | Cl(25)-C(22)-C(23) | 119.4 | N(26)-C(25)-C(16) | 178.3 |  |  |
| C(25)-C(24)-C(23) | 118.8 | O(25)-C(22)-C(23) | 124.6 | Cl(25)-C(22)-C(21) | 119.3 | C(23)-C(24)-C(1) | 119.1 |  |  |
| N(26)-C(23)-C(24) | 120.9 | O(25)-C(22)-C(21) | 115.5 | C(23)-C(22)-C(21) | 121.3 | C(24)-C(23)-C(22) | 118.8 |  |  |
| N(26)-C(23)-C(22) | 116.6 | C(23)-C(22)-C(21) | 119.9 | C(22)-C(21)-C(20) | 119.3 | N(27)-C(22)-C(23) | 121.0 |  |  |
| C(24)-C(23)-C(22) | 122.4 | C(22)-C(21)-C(20) | 120.2 | C(21)-C(20)-C(1) | 119.4 | N(27)-C(22)-C(21) | 116.6 |  |  |
| C(23)-C(22)-C(21) | 118.7 | C(21)-C(20)-C(1) | 119.5 | C(17)-N(18)-N(14) | 104.9 | C(23)-C(22)-C(21) | 122.5 |  |  |
| C(22)-C(21)-C(1) | 119.2 | C(17)-N(18)-N(14) | 104.9 | N(18)-C(17)-C(16) | 112.8 | C(22)-C(21)-C(20) | 118.7 |  |  |
| C(17)-N(18)-N(14) | 108.6 | N(18)-C(17)-C(16) | 112.8 | C(26)-C(16)-C(17) | 129.5 | C(21)-C(20)-C(1) | 119.2 |  |  |
| C(19)-C(17)-N(18) | 121.6 | C(27)-C(16)-C(17) | 129.5 | C(26)-C(16)-C(15) | 125.6 | C(17)-N(18)-N(14) | 104.9 |  |  |
| C(19)-C(17)-C(16) | 125.4 | C(27)-C(16)-C(15) | 125.6 | C(17)-C(16)-C(15) | 104.9 | N(18)-C(17)-C(16) | 112.8 |  |  |
| N(18)-C(17)-C(16) | 112.9 | C(17)-C(16)-C(15) | 104.9 | N(19)-C(15)-C(16) | 129.3 | C(25)-C(16)-C(17) | 129.5 |  |  |
| C(17)-C(16)-C(15) | 102.3 | N(19)-C(15)-C(16) | 129.3 | N(19)-C(15)-N(14) | 124.6 | C(25)-C(16)-C(15) | 125.5 |  |  |
| O(20)-C(15)-C(16) | 128.3 | N(19)-C(15)-N(14) | 124.7 | C(16)-C(15)-N(14) | 105.9 | C(17)-C(16)-C(15) | 104.9 |  |  |
| O(20)-C(15)-N(14) | 127.7 | C(16)-C(15)-N(14) | 106.0 | N(18)-N(14)-C(15) | 111.4 | N(19)-C(15)-C(16) | 129.4 |  |  |
| C(16)-C(15)-N(14) | 104.0 | N(18)-N(14)-C(15) | 111.4 | N(18)-N(14)-C(12) | 123.0 | N(19)-C(15)-N(14) | 124.6 |  |  |
| N(18)-N(14)-C(15) | 112.2 | N(18)-N(14)-C(12) | 123.1 | C(15)-N(14)-C(12) | 125.3 | C(16)-C(15)-N(14) | 105.9 |  |  |
| N(18)-N(14)-C(12) | 121.3 | C(15)-N(14)-C(12) | 125.2 | N(14)-C(12)-O(13) | 118.1 | N(18)-N(14)-C(15) | 111.5 |  |  |
| C(15)-N(14)-C(12) | 126.0 | N(14)-C(12)-O(13) | 117.9 | N(14)-C(12)-C(9) | 120.4 | N(18)-N(14)-C(12) | 122.9 |  |  |
| N(14)-C(12)-O(13) | 119.8 | N(14)-C(12)-C(9) | 120.5 | O(13)-C(12)-C(9) | 121.5 | C(15)-N(14)-C(12) | 125.4 |  |  |
| N(14)-C(12)-C(9) | 118.4 | O(13)-C(12)-C(9) | 121.6 | C(10)-C(11)-C(6) | 120.1 | N(14)-C(12)-O(13) | 118.4 |  |  |
| O(13)-C(12)-C(9) | 121.8 | C(10)-C(11)-C(6) | 120.2 | C(11)-C(10)-C(9) | 120.9 | N(14)-C(12)-C(9) | 120.2 |  |  |
| C(10)-C(11)-C(6) | 119.8 | C(11)-C(10)-C(9) | 120.9 | C(12)-C(9)-C(10) | 125.7 | O(13)-C(12)-C(9) | 121.4 |  |  |
| C(11)-C(10)-C(9) | 120.9 | C(12)-C(9)-C(10) | 125.8 | C(12)-C(9)-C(8) | 115.6 | C(10)-C(11)-C(6) | 120.0 |  |  |
| C(12)-C(9)-C(10) | 124.6 | C(12)-C(9)-C(8) | 115.6 | C(10)-C(9)-C(8) | 118.5 | C(11)-C(10)-C(9) | 120.8 |  |  |
| C(12)-C(9)-C(8) | 116.4 | C(10)-C(9)-C(8) | 118.5 | C(9)-C(8)-C(7) | 120.9 | C(12)-C(9)-C(10) | 125.4 |  |  |
| C(10)-C(9)-C(8) | 118.8 | C(9)-C(8)-C(7) | 120.9 | C(8)-C(7)-C(6) | 120.2 | C(12)-C(9)-C(8) | 115.7 |  |  |
| C(9)-C(8)-C(7) | 120.7 | C(8)-C(7)-C(6) | 120.3 | C(11)-C(6)-C(7) | 119.3 | C(10)-C(9)-C(8) | 118.7 |  |  |
| C(8)-C(7)-C(6) | 120.1 | C(11)-C(6)-C(7) | 119.2 | C(11)-C(6)-N(5) | 121.7 | C(9)-C(8)-C(7) | 120.8 |  |  |
| C(11)-C(6)-C(7) | 119.7 | C(11)-C(6)-N(5) | 121.7 | C(7)-C(6)-N(5) | 118.8 | C(8)-C(7)-C(6) | 120.1 |  |  |
| C(11)-C(6)-N(5) | 121.0 | C(7)-C(6)-N(5) | 119.0 | C(6)-N(5)-S(2) | 125.0 | C(11)-C(6)-C(7) | 119.5 |  |  |
| C(7)-C(6)-N(5) | 119.3 | C(6)-N(5)-S(2) | 124.6 | N(5)-S(2)-O(4) | 107.2 | C(11)-C(6)-N(5) | 121.4 |  |  |
| C(6)-N(5)-S(2) | 123.7 | N(5)-S(2)-O(4) | 106.9 | N(5)-S(2)-O(3) | 103.2 | C(7)-C(6)-N(5) | 119.0 |  |  |
| N(5)-S(2)-O(4) | 107.4 | N(5)-S(2)-O(3) | 102.8 | N(5)-S(2)-C(1) | 106.1 | C(6)-N(5)-S(2) | 124.6 |  |  |
| N(5)-S(2)-O(3) | 104.0 | N(5)-S(2)-C(1) | 106.3 | O(4)-S(2)-O(3) | 123.1 | N(5)-S(2)-O(4) | 107.5 |  |  |
| N(5)-S(2)-C(1) | 105.9 | O(4)-S(2)-O(3) | 122.9 | O(4)-S(2)-C(1) | 107.8 | N(5)-S(2)-O(3) | 103.7 |  |  |
| O(4)-S(2)-O(3) | 123.3 | O(4)-S(2)-C(1) | 108.2 | O(3)-S(2)-C(1) | 108.2 | N(5)-S(2)-C(1) | 105.7 |  |  |
| O(4)-S(2)-C(1) | 107.3 | O(3)-S(2)-C(1) | 108.6 | C(24)-C(1)-C(20) | 121.3 | O(4)-S(2)-O(3) | 123.3 |  |  |
| O(3)-S(2)-C(1) | 107.8 | C(24)-C(1)-C(20) | 120.8 | C(24)-C(1)-S(2) | 119.7 | O(4)-S(2)-C(1) | 107.5 |  |  |
| C(25)-C(1)-C(21) | 121.7 | C(24)-C(1)-S(2) | 119.9 | C(20)-C(1)-S(2) | 119.0 | O(3)-S(2)-C(1) | 107.9 |  |  |
| C(25)-C(1)-S(2) | 119.4 | C(20)-C(1)-S(2) | 119.4 |  |  | C(24)-C(1)-C(20) | 121.8 |  |  |
| C(21)-C(1)-S(2) | 118.9 |  |  |  |  | C(24)-C(1)-S(2) | 119.4 |  |  |
|  |  |  |  |  |  | C(20)-C(1)-S(2) | 118.8 |  |  |

**Table S4.** Mulliken’s atomic charges of explored hybrids.

|  | **5a** |  | **5b** |  | **5c** |  | **6a** |  | **6b** |  | **6c** |  | **7a** |  | **7b** |  | **7c** |
| --- | --- | --- | --- | --- | --- | --- | --- | --- | --- | --- | --- | --- | --- | --- | --- | --- | --- |
| C(1) | -0.141 | C(1) | -0.114 | C(1) | -0.120 | C(1) | -0.140 | C(1) | -0.113 | C(1) | -0.120 | C(1) | -0.139 | C(1) | -0.112 | C(1) | -0.119 |
| S(2) | 1.279 | S(2) | 1.282 | S(2) | 1.288 | S(2) | 1.277 | S(2) | 1.282 | S(2) | 1.288 | S(2) | 1.284 | S(2) | 1.289 | S(2) | 1.292 |
| O(3) | -0.588 | O(3) | -0.585 | O(3) | -0.580 | O(3) | -0.586 | O(3) | -0.584 | O(3) | -0.578 | O(3) | -0.585 | O(3) | -0.582 | O(3) | -0.576 |
| O(4) | -0.590 | O(4) | -0.584 | O(4) | -0.578 | O(4) | -0.592 | O(4) | -0.585 | O(4) | -0.580 | O(4) | -0.589 | O(4) | -0.583 | O(4) | -0.577 |
| N(5) | -0.756 | N(5) | -0.758 | N(5) | -0.761 | N(5) | -0.755 | N(5) | -0.757 | N(5) | -0.761 | N(5) | -0.760 | N(5) | -0.762 | N(5) | -0.764 |
| C(6) | 0.450 | C(6) | 0.443 | C(6) | 0.445 | C(6) | 0.437 | C(6) | 0.435 | C(6) | 0.440 | C(6) | 0.470 | C(6) | 0.469 | C(6) | 0.460 |
| C(7) | -0.381 | C(7) | -0.377 | C(7) | -0.376 | C(7) | -0.371 | C(7) | -0.370 | C(7) | -0.371 | C(7) | -0.384 | C(7) | -0.384 | C(7) | -0.380 |
| C(8) | -0.300 | C(8) | -0.301 | C(8) | -0.301 | C(8) | -0.304 | C(8) | -0.304 | C(8) | -0.304 | C(8) | -0.291 | C(8) | -0.291 | C(8) | -0.292 |
| C(9) | 0.142 | C(9) | 0.148 | C(9) | 0.150 | C(9) | 0.160 | C(9) | 0.164 | C(9) | 0.165 | C(9) | 0.122 | C(9) | 0.127 | C(9) | 0.133 |
| C(10) | -0.256 | C(10) | -0.256 | C(10) | -0.255 | C(10) | -0.256 | C(10) | -0.255 | C(10) | -0.253 | C(10) | -0.256 | C(10) | -0.256 | C(10) | -0.257 |
| C(11) | -0.363 | C(11) | -0.356 | C(11) | -0.357 | C(11) | -0.347 | C(11) | -0.346 | C(11) | -0.352 | C(11) | -0.379 | C(11) | -0.377 | C(11) | -0.365 |
| C(12) | 0.342 | C(12) | 0.341 | C(12) | 0.340 | C(12) | 0.347 | C(12) | 0.346 | C(12) | 0.345 | C(12) | 0.394 | C(12) | 0.394 | C(12) | 0.393 |
| O(13) | -0.378 | O(13) | -0.376 | O(13) | -0.374 | O(13) | -0.334 | O(13) | -0.332 | O(13) | -0.331 | O(13) | -0.421 | O(13) | -0.419 | O(13) | -0.415 |
| N(14) | -0.066 | N(14) | -0.065 | N(14) | -0.064 | N(14) | -0.153 | N(14) | -0.152 | N(14) | -0.151 | N(14) | -0.075 | N(14) | -0.074 | N(14) | -0.073 |
| C(15) | 0.228 | C(15) | 0.229 | C(15) | 0.229 | C(15) | 0.327 | C(15) | 0.328 | C(15) | 0.329 | C(15) | 0.292 | C(15) | 0.293 | C(15) | 0.294 |
| C(16) | -0.439 | C(16) | -0.437 | C(16) | -0.436 | C(16) | -0.581 | C(16) | -0.581 | C(16) | -0.582 | C(16) | -0.003 | C(16) | -0.002 | C(16) | -0.001 |
| C(17) | 0.176 | C(17) | 0.176 | C(17) | 0.177 | C(17) | 0.206 | C(17) | 0.207 | C(17) | 0.207 | C(17) | -0.234 | C(17) | -0.233 | C(17) | -0.231 |
| N(18) | -0.246 | N(18) | -0.246 | N(18) | -0.246 | N(18) | -0.175 | N(18) | -0.175 | N(18) | -0.176 | N(18) | -0.199 | N(18) | -0.200 | N(18) | -0.201 |
| C(19) | -0.680 | C(19) | -0.679 | C(19) | -0.679 | C(19) | -0.671 | C(19) | -0.670 | C(19) | -0.669 | N(19) | -0.736 | N(19) | -0.736 | N(19) | -0.736 |
| C(20) | -0.664 | C(20) | -0.664 | C(20) | -0.663 | O(20) | -0.294 | O(20) | -0.292 | O(20) | -0.290 | C(20) | -0.252 | C(20) | -0.262 | C(20) | -0.247 |
| C(21) | -0.256 | C(21) | -0.266 | C(21) | -0.249 | C(21) | -0.257 | C(21) | -0.266 | C(21) | -0.248 | C(21) | -0.322 | C(21) | -0.200 | C(21) | -0.303 |
| C(22) | -0.322 | C(22) | -0.200 | C(22) | -0.303 | C(22) | -0.321 | C(22) | -0.199 | C(22) | -0.303 | C(22) | 0.439 | C(22) | -0.020 | C(22) | 0.271 |
| C(23) | 0.438 | C(23) | -0.020 | C(23) | 0.270 | C(23) | 0.438 | C(23) | -0.020 | C(23) | 0.270 | C(23) | -0.373 | C(23) | -0.201 | C(23) | -0.303 |
| C(24) | -0.372 | C(24) | -0.201 | C(24) | -0.303 | C(24) | -0.372 | C(24) | -0.201 | C(24) | -0.302 | C(24) | -0.227 | C(24) | -0.218 | C(24) | -0.200 |
| C(25) | -0.227 | C(25) | -0.217 | C(25) | -0.198 | C(25) | -0.230 | C(25) | -0.219 | C(25) | -0.199 | O(25) | -0.325 | Cl(25) | -0.054 | C(25) | -0.061 |
| O(26) | -0.326 | Cl(26) | -0.059 | N(26) | 0.326 | O(26) | -0.325 | Cl(26) | -0.058 | N(26) | 0.326 | C(26) | -0.474 | C(26) | -0.062 | N(26) | -0.181 |
| C(27) | -0.474 |  |  | O(27) | -0.311 | C(27) | -0.473 | H(27) | 0.384 | O(27) | -0.309 | C(27) | -0.062 | N(27) | -0.183 | N(27) | 0.326 |
|  |  |  |  | O(28) | -0.309 |  |  |  |  | O(28) | -0.310 | N(28) | -0.185 |  |  | O(28) | -0.309 |

**Table S5.** *In vitro* inhibitory activities of the synthesized pyrazole-sulfonamide hybrids against DHFR enzyme.

| **Hybrids** | **IC_50_ (Mean ± SEM) (µM)** |
| --- | --- |
|  | **DHFR** |
| **5a** | 2.66±0.07 |
| **5b** | 2.03±0.11 |
| **5c** | 1.88±0.39 |
| **6a** | 2.91±0.21 |
| **6b** | 3.84±0.50 |
| **6c** | 3.29±0.42 |
| **7a** | 1.46±0.18 |
| **7b** | 1.79±0.06 |
| **7c** | 1.37±0.33 |
| **Methotrexate** | 1.19±0.30 |

N.B^:^ IC_50_ values are the mean ± SD of three separate experiments

**Table S6**. Docking results of the synthesized pyrazole-sulphonamide hybrids with PDB: 3TZF.

| **Hybrids** | **Binding energy** | **RMSD** | **ligand bindings with**  **the amino-acid residues** | **Types of**  **Interactions** | **Distance (Å)** |
| --- | --- | --- | --- | --- | --- |
| **5a** | -5.2683 | 1.9687 | N 5 of the sulphonamide group with Glu73  The pyrazole-ring with Leu37 | H-donor  pi-H | 3.05  3.75 |
| **5b** | -5.8069 | 1.5105 | O 39 of the sulphonamide group with Lys146 | H-acceptor | 3.18 |
| **5c** | -5.6274 | 1.3443 | O 4 of the sulphonamide group with Met1  O 4 of the sulphonamide group with His2  The phenyl-ring with Asn180 | H-acceptor  H-acceptor  pi-H | 3.52  3.10  4.26 |
| **6a** | -6.5129 | 1.5461 | O 24 of the sulphonamide group with Thr62  O 33 of the amide group with Ser222  The aniline-ring with Lys221 | H-acceptor  H-acceptor  pi-H | 3.00  2,.87  4.20 |
| **6b** | -4.2781 | 1.6556 | O 23 of the sulphonamide group with Asp11 | H-acceptor | 3.02 |
| **6c** | -5.1452 | 1.8051 | O 19 of the sulphonamide group with Asp11 | H-acceptor | 3.21 |
| **7a** | -5.8829 | 1.6474 | N 4 of the sulphonamide group with Asn180  O 2 of the sulphonamide group with Asn180  N 28 of the nitrile group with Glu211  The Anisyl-ring with Thr4 | H-donor  H-acceptor  H-acceptor  pi-H | 3.40  3.16  3.68  4.86 |
| **7b** | -5.7566 | 1.5774 | N 15 of the nitrile group with Gln121  N 24 of the pyrazole-ring with Gly143  The phenyl-ring with Arg163 | H‐acceptor  H-acceptor  pi-H | 3.28  3.39  3.90 |
| **7c** | -5.9413 | 1.3003 | N 29 of the nitrile group with Thr4 | H‐acceptor | 3.25 |
| **Methotrexate** | -6.4743 | 1.4710 | N 10 of the amino group with Glu 261  The pyrimidine-ring with Lys260 | H-donor  pi-H | 3.31  4.55 |

**Table S7**. SwissADME results of the synthesized pyrazole-sulphonamide hybrids.

| **No** | **M. Wt.** | **Heavy**  **atoms** | **RT** | **HA** | **HD** | **TPSA** | **iLOGP** | **ESOL**  **Class** | **GI**  **absorption** | **BBB**  **permeant** | **Pgp**  **substrate** | **Lipinski**  **violations** | **Bio-available**  **Score** |
| --- | --- | --- | --- | --- | --- | --- | --- | --- | --- | --- | --- | --- | --- |
| **5a** | 423.45 | 31 | 5 | 6 | 1 | 148.52 | 2.22 | Moderate | Low | No | No | 0 | 0.55 |
| **5b** | 453.48 | 33 | 6 | 7 | 1 | 157.75 | 2.66 | Moderate | Low | No | No | 0 | 0.55 |
| **5c** | 468.45 | 34 | 6 | 8 | 1 | 194.34 | 1.57 | Moderate | Low | No | No | 1 | 0.55 |
| **6a** | 440.48 | 32 | 6 | 6 | 1 | 141.8 | 2.26 | Moderate | Low | No | No | 0 | 0.55 |
| **6b** | 470.5 | 34 | 7 | 7 | 1 | 151.03 | 2.66 | Moderate | Low | No | No | 0 | 0.55 |
| **6c** | 485.47 | 35 | 7 | 8 | 1 | 187.62 | 1.8 | Moderate | Low | No | No | 1 | 0.55 |
| **7a** | 374.42 | 27 | 5 | 5 | 2 | 124.79 | 2.3 | Moderate | High | No | No | 0 | 0.55 |
| **7b** | 404.45 | 29 | 6 | 6 | 2 | 134.02 | 2.46 | Moderate | Low | No | No | 0 | 0.55 |
| **7c** | 419.42 | 30 | 6 | 7 | 2 | 170.61 | 1.6 | Moderate | Low | No | No | 0 | 0.55 |

Where, M.wt: molecular weight, M iLog P: lipophilicity parameter, HBA: hydrogen bond acceptor, HBD: hydrogen bond donor, TPSA: topological polar surface area, RT: rotatable bonds.

|  |  |
| --- | --- |
| **6a** | **6b** |
|  |  |
| **6c** | **7a** |
|  |  |
| **7b** | **7c** |

**Figure S1**. The optimized structures of derivatives **6a-c** and **7a-c**

| **HOMO** | **LUMO** |
| --- | --- |
|  |  |
| **5b** | |
|  |  |
| **6a** | |
|  |  |
| **6b** | |
|  |  |
| **6c** | |
|  |  |
| **7b** | |

**Figure S2**. The 3D-plot of FMO’s for **5b**, **6a-c** and **7c** compounds.

| **2D** | **3D** | **Surface Map** |
| --- | --- | --- |
|  |  |  |
| **5a** | | |
|  |  |  |
| **5b** | | |
|  |  |  |
| **5c** | | |
|  |  |  |
| **6b** | | |
|  |  |  |
| **6c** | | |
|  |  |  |
| **Methotrexate** | | |

**Figure S3**. Docking images of hybrids **5a-c**, **6b**, **6c**, and methotrexate with ID: 3TZF.

| a | | | b |
| --- | --- | --- | --- |
|  |  |  |  |
| **5a** | **5b** | **5c** |  |
|  |  |  |  |
| **6a** | **6b** | **6c** |  |
|  |  |  |  |
| **7a** | **7b** | **7c** |  |

**Figure S4.(I; 5a-5c: II; 6a-6c: III; 7a-7c)** Radar chart and boiled egg of the synthesized pyrazole-sulphonamide hybrids.

**Figure S4.** ^1^H-NMR spectrum of compound **5a**.

**Figure S4.** ^13^C-NMR spectrum of compound **5a**.

**Figure S4.** ^1^H-NMR spectrum of compound **5b**.

**Figure S4.** ^13^C-NMR spectrum of compound **5b**.

**Figure S4.** ^1^H-NMR spectrum of compound **5c**.

**Figure S4.** ^13^C-NMR spectrum of compound **5c**.

**Figure S4.** ^1^H-NMR spectrum of compound **6a**.

**Figure S4.** ^13^C-NMR spectrum of compound **6a**.

**Figure S4.** ^1^H-NMR spectrum of compound **6b**.

**Figure S4.** ^13^C-NMR spectrum of compound **6b**.

**Figure S4.** ^1^H-NMR spectrum of compound **6c**.

**Figure S4.** ^13^C-NMR spectrum of compound **6c**.

**Figure S4.** ^1^H-NMR spectrum of compound **7a**.

**Figure S4.** ^13^C-NMR spectrum of compound **7a**.

**Figure S4.** ^1^H-NMR spectrum of compound **7b**.

**Figure S4.** ^13^C-NMR spectrum of compound **7b**.

**Figure S4.** ^1^H-NMR spectrum of compound **7c**.

**Figure S4.** ^13^C-NMR spectrum of compound **7c**.
